# Construction and Property Investigation of Serial Pillar[5]arene-Based [1]Rotaxanes

**DOI:** 10.3389/fchem.2022.908773

**Published:** 2022-06-07

**Authors:** Longtao Ma, Ying Han, Chaoguo Yan, Tingting Chen, Yang Wang, Yong Yao

**Affiliations:** ^1^ School of Chemistry and Chemical Engineering, Yangzhou University, Yangzhou, China; ^2^ School of Chemistry and Chemical Engineering, Nantong University, Nantong, China

**Keywords:** pillar[5]arene, mechanically interlocked molecule, self-inclusion, host–guest interaction, [1]rotaxanes

## Abstract

Although the construction and application of pillar[5]arene-based [1]rotaxanes have been extensively studied, the types of stoppers for them are limited. In this work, we designed and prepared three series of pillar[5]arene-based [1]rotaxanes (**P5[1]Rs**) with pentanedione derivatives, azobenzene derivatives, and salicylaldehyde derivatives as the stoppers, respectively. The obtained **P5[1]Rs** were fully characterized by NMR (^1^H, ^13^C, and 2D), mass spectra, and single-crystal X-ray analysis. We found that the synergic C–H···π, C–H···O interactions and N–H···O, O–H···N hydrogen bonding are the key to the stability of [1]rotaxanes. This work not only enriched the diversity of pillar[n]arene family but also gave a big boost to the pillar[n]arene-based mechanically interlocked molecules

## Introduction

**Scheme 1:**
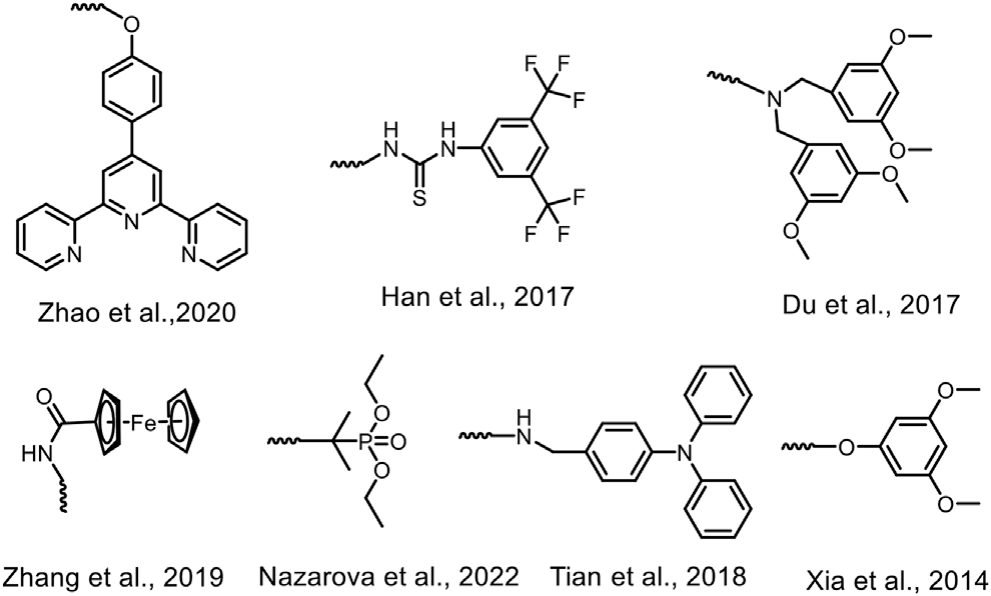
Chemical structures of different types of reported stoppers for the formation of [1]rotaxanes.

Mechanically interlocked molecules (MIMs), mainly including knots, rotaxanes, and catenanes, are a new type of fascinating molecules that contain mechanical bonds and allow for large movements at the molecular level (Hu et al., 2017; Zhou et al., 2020). MIMs cannot be separated without breaking the participating covalent bonds due to the mechanical bonds forming an entanglement in space. During the past decades, MIMs have drawn a tremendous interest not only due to their smart architectures but also due to their potential applications in various fields (Sluysmans et al., 2019; Cornelissen et al., 2021; David et al., 2021). [1]Rotaxanes, which are composed of “T”-shaped axles and macrocyclic wheels and the axles are threaded by their own wheels and connected by covalent bonds, are considered as the fundamental supramolecular systems for the construction of diverse MIMs (Tian et al., 2018; Scheme 1). It is known that there is a huge challenge for the efficient synthesis of [1]rotaxanes due to their subtle structures.

Pillar[5]arenes, first reported by Ogoshi et al. (2008), are considered the fifth generation of classical macrocyclic compounds (An et al., 2021) after crown ethers (Liu et al., 2017; Wu et al., 2020; Bai et al., 2022), cyclodextrins (Bolton et al., 2020; Sugita et al., 2022), calixarenes (An et al., 2019; Li B. et al., 2020; Zhang et al., 2021), and cucurbiturils (Chernikova et al., 2020; Zhang et al., 2022). Pillar[5]arenes are composed of five hydroquinone derivatives, which are linked by –CH_2_– at the *2,5*-positions (Shi et al., 2011; Strutt et al., 2012). During the past 14 years, great developments have been made in the synthesis (Yao et al., 2012; Fan et al., 2016; Wu et al., 2021), host–guest properties (Ogoshi et al., 2015; Zhou et al., 2016; Lu et al., 2022), self-assembly (Guo et al., 2020; Cai et al., 2021a; Cao et al., 2021; Wang et al., 2021; Wu et al., 2022), and applications (Tan et al., 2015; Li L. et al., 2020; Cai et al., 2021b; Guo et al., 2021; Yan et al., 2021) of pillar[5]arenes. Pillar[5]arene-based rotaxanes (**P5Rs**) have also attracted much attention. In 2011, Prof. Stoddart and *co*-workers prepared the first P5R in two steps. First, a host–guest complex was fabricated between DMpillar[5]arene and 1,8-diaminooctane. Then, the stopper 3,5-di-tertbutylbenzaldehyde reacted with the amino groups on the guest to block the cavity to form **P5R** (Strutt et al., 2011). After that, numerous types of **P5[1]Rs** with various longer axels and different sizes of stoppers have been designed and synthesized (Han et al., 2017; Nazarova et al., 2022; Scheme 1). For example, in 2014, Xia and *co*-workers designed and synthesized a **P5[1]R** with the yield up to 73% in three steps (Xia et al., 2014; Scheme 1). Firstly, they prepared a monocarboxylic acid-functionalized pillar[5]arene through the *co*-oligomerization reaction and a linear guest with a stopper on one side and a primary amine group on the other side. Next, due to the C–H⋯π interactions, the alkyl chain on the guest is passed through the cavity of the pillar[5]arene, where the primary amine group and the carboxylic acid group are expected to form an ion pair complex. Lastly, the primary amine group on the guest and the carboxylic acid group on pillar[5]arene reacted *via* Schiff-base formation to afford **P5[1]R**. Besides, our groups have constructed a couple of **P5[1]Rs** from their parent pseudo[1]rotaxanes in recent years through aldoamine condensation (Zhang et al., 2019; Zhao et al., 2020; Scheme 1).

Although great progress has been made in the research of **P5[1]Rs** (Du et al., 2017; Scheme 1), the species of **P5[1]Rs**, especially the types of the stoppers, are not abundant enough. In this work, we designed and prepared three series of **P5[1]Rs** with different stoppers from their parent pseudo[1]rotaxanes *via* the “threading-followed-by-stoppering” method. The obtained **P5[1]Rs** were characterized by various technologies, such as ^1^H NMR, ^13^C NMR, 2D-NOESY spectra, and X-ray single-crystal diffraction.

## Experimental Section

### Syntheses of Pillar[5]arene-Based [1]Rotaxanes

**Scheme 2:**
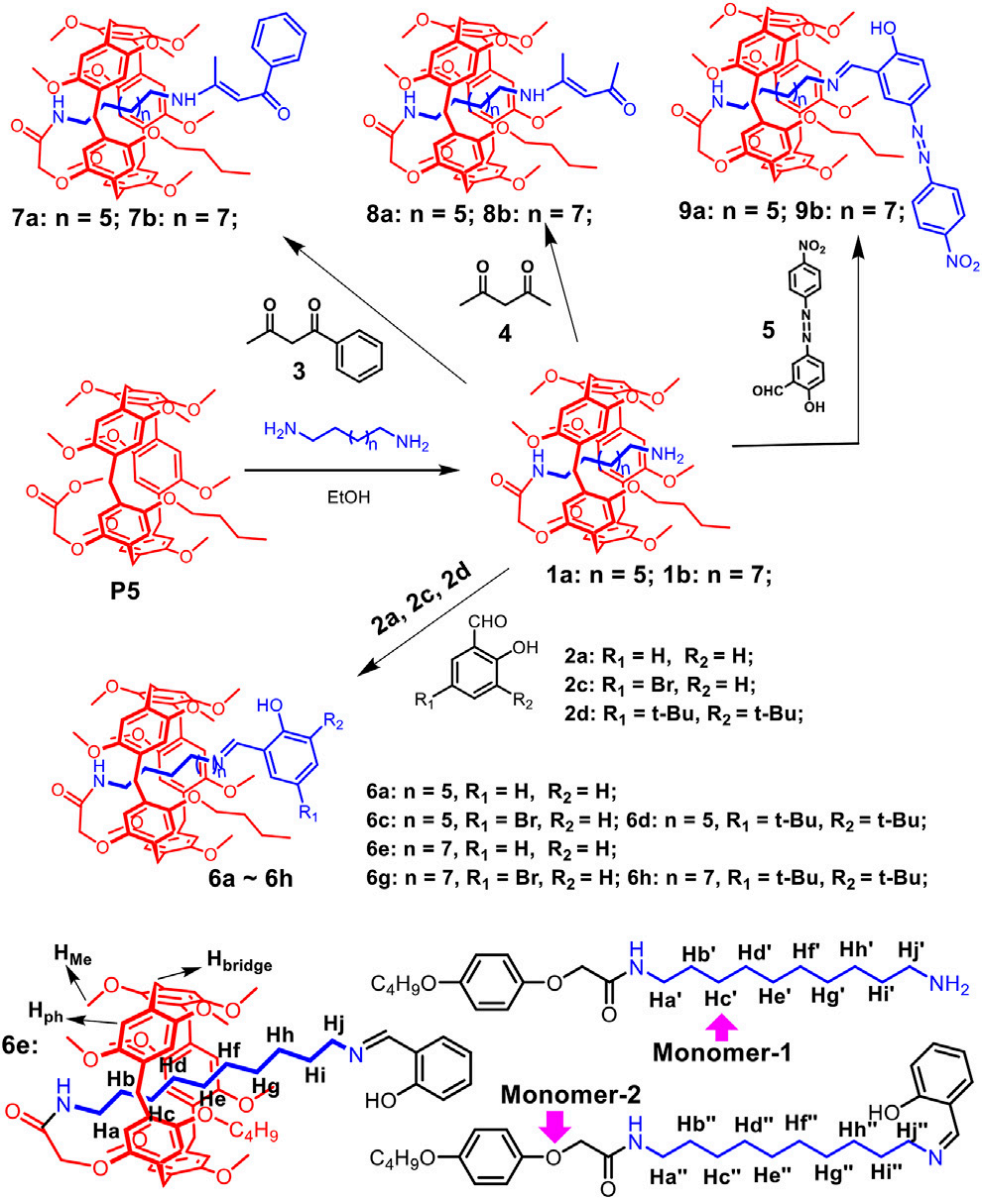
Synthetic route to pillar[5]arene-based [1]rotaxanes and the chemical structures of **6e** and **monomers**.

Based on previous reports (Zhang et al., 2019), pseudo[1]rotaxanes **1a** and **1b** were prepared directly from **P5** and alkyl-diamine in CH_3_CH_2_OH (Scheme 2, and Supplementary Figures S1–S6). Then, **P5[1]Rs** were successfully synthesized by **1a** or **1b** reacted with the stoppers (**2**, **3**, **4**, **5**) under the catalysis of CH_3_COOH. [1]Rotaxene **6e** is taken as an example. Compound **1b** (0.198 g, 0.2 mmol), stopper **2a** (0.025 g, 0.2 mmol), and 0.1 ml CH_3_COOH were stirred in 10 ml of dry CH_3_CH_2_OH for 12 h at 80°C. The reaction solvent was evaporated, and the residue was purified by flash column chromatography on silica gel (CH_2_Cl_2_/CH_3_OH, *v*/*v* 20:1) to give **6e** as a yellow solid (0.167 g). Other **P5[1]Rs** were prepared according to the same method (Scheme 2).

**6e**: light yellow solid, yield 49%. m.p. 85–86°C; ^1^H NMR (Supplementary Figure S16) (400 MHz, CDCl_3_) δ 13.57 (s, 1H, OH), 8.39 (s, 1H, CH), 7.36–7.31 (m, 1H, ArH), 7.28 (m, 1H, ArH), 6.99 (d, *J* = 8.3 Hz, 1H, ArH), 6.91 (m, 6H, ArH), 6.86–6.82 (t, *J* = 2 Hz, 4H, ArH), 6.71 (s, 1H, ArH), 5.06 (s, 1H, NH), 4.57 (s, 2H, CH_2_), 3.88 (d, *J* = 7.0 Hz, 2H, CH_2_), 3.79–3.71 (m, 34H, 8CH_3_, 5CH_2_), 3.64 (t, *J* = 6.7 Hz, 2H, CH_2_), 2.45 (s, 2H, CH_2_), 1.86–1.78 (d, *J* = 2 Hz, 2H,CH_2_), 1.73 (t, *J* = 7.8 Hz, 2H, CH_2_), 1.61 (d, *J* = 7.4 Hz, 2H, CH_2_), 1.40 (t, *J* = 7.9 Hz, 2H, CH_2_), 1.19 (s, 2H, CH_2_), 1.02 (t, *J* = 7.4 Hz, 3H, CH_3_), 0.77 (s, 2H, CH_2_), −0.08 (s, 2H, CH_2_), −1.38 (s, 4H, CH_2_), −2.36 (s, 2H,CH_2_); ^13^C NMR (Supplementary Figure S17) (101 MHz, CDCl_3_) δ 167.2, 164.6, 161.3, 150.8, 150.4, 150.3, 150.3, 150.2, 150.0, 147.0, 132.2, 131.1, 129.4, 129.0, 128.3, 128.2, 128.1, 127.9, 127.8, 127.8, 127.1, 126.8, 118.8, 118.6, 117.0, 114.7, 113.9, 113.6, 113.3, 112.7, 112.4, 67.8, 65.8, 59.7, 55.5, 55.4, 55.3, 55.1, 38.0, 32.1, 31.3, 30.8, 30.6, 30.2, 29.7, 29.3, 28.9, 28.7, 28.3, 28.1, 26.5, 23.6, 19.6, 14.1; HRMS (ESI) (Supplementary Figure S18) calcd. for C_66_H_82_N_2_O_12_([M + Na]^+^): 1117.5765, found: 1117.5770.

**6a**: light yellow solid, yield 76%. m.p. 106–107°C; ^1^H NMR (Supplementary Figure S7) (400 MHz, CDCl_3_) δ 13.47 (s, 1H, OH), 8.38 (s, 1H, CH), 7.35 (t, *J* = 8 Hz, 1H, ArH), 7.28 (d, *J* = 6 Hz, 1H, ArH), 7.00 (d, *J* = 8.4 Hz, 1H, ArH), 6.92 (m, 5H, ArH), 6.88 (s, 1H, ArH), 6.84 (m, 4H, ArH), 6.73 (s, 1H, ArH), 5.09 (s, 1H, NH), 4.57 (s, 2H, CH_2_), 3.88 (d, *J* = 3.6 Hz, 2H, CH_2_), 3.79–3.69 (m, 34H, 8OCH_3_, 5CH_2_), 3.58 (d, *J* = 6.6 Hz, 2H, CH_2_), 2.48 (s, 2H, CH_2_), 1.82 (t, *J* = 14.7 Hz, 2H, CH_2_), 1.61 (s, 2H, CH_2_), 1.53 (d, *J* = 11.2 Hz, 2H, CH_2_), 0.99 (t, *J* = 7.4 Hz, 3H, CH_3_), 0.81 (s, 2H, CH_2_), −0.10 (s, 2H, CH_2_), −1.35 (s, 4H, CH_2_), −2.31 (s, 2H, CH_2_); ^13^C NMR (Supplementary Figure S8) (101 MHz, CDCl_3_) δ 167.3, 164.4, 161.2, 150.8, 150.4, 150.3, 150.2, 150.1, 150.0, 132.3, 131.0, 130.0, 129.0, 128.4, 128.3, 128.0, 127.9, 127.3, 126.9,118.7, 118.6, 117.1, 115.0, 114.1, 113.7, 113.5, 113.4, 112.8, 112.4, 68.1, 65.9, 60.4, 55.6, 55.5, 55.4, 55.3, 55.2, 38.1, 32.1, 31.9, 30.2, 29.2, 28.9, 28.7, 28.5, 28.3, 28.2, 26.4, 23.6, 19.7, 14.1; HRMS (ESI) (Supplementary Figure S9) calcd. for C_64_H_78_N_2_O_12_([M + Na]^+^): 1089.5452, found: 1089.5437.

**6c**: light yellow solid, yield 89%. m.p. 92–93°C; ^1^H NMR (Supplementary Figure S10) (400 MHz, CDCl_3_) δ 13.52 (s, 1H, OH), 8.31 (s, 1H, CH), 7.44–7.38 (m, 2H, ArH), 6.94–6.87 (m, 6H, ArH), 6.85–6.82 (t, *J* = 4 Hz, 4H, ArH), 6.72 (s, 1H, ArH), 5.09 (s, 1H, NH), 4.57 (s, 2H, CH_2_), 3.90–3.85 (m, 2H, CH_2_), 3.80–3.70 (m, 34H, 8OCH_3_,5CH_2_), 3.58 (t, *J* = 7.2 Hz, 2H, CH_2_), 2.48 (s, 2H, CH_2_), 1.81 (d, *J* = 15.5 Hz, 2H, CH_2_), 1.59 (t, *J* = 7.5 Hz, 2H, CH_2_), 1.50 (t, *J* = 7.3 Hz, 2H, CH_2_), 0.99 (t, *J* = 7.4 Hz, 3H, CH_3_), 0.80 (s, 2H, CH_2_), −0.11 (s, 2H, CH_2_), −1.34 (s, 4H, CH_2_), −2.28 (s, 2H, CH_2_); ^13^C NMR (Supplementary Figure S11) (101 MHz, CDCl_3_) δ 167.3, 163.1, 160.3, 150.8, 150.4, 150.4, 150.3, 150.3, 150.2, 150.0, 147.1, 134.9, 133.1, 129.5, 129.1, 128.4, 128.3, 128.2, 128.1, 127.9, 127.9, 127.3, 126.9, 120.1, 119.1, 115.0, 114.1, 113.8, 113.6, 113.5, 112.7, 112.4, 110.0, 68.1, 65.9, 60.3, 55.7, 55.6, 55.4, 55.3, 55.1, 38.1, 32.0, 31.8, 30.2, 29.3, 29.1, 28.8, 28.7, 28.4, 28.3, 28.1, 26.3, 23.6, 19.6, 14.1; HRMS (ESI) (Supplementary Figure S12) calcd. for C_64_H_77_BrN_2_O_12_([M + Na]^+^): 1167.4558, found: 1167.4537.

**6d**: yellow solid, yield 47%. m.p. 108–109°C; ^1^H NMR (Supplementary Figure S13) (400 MHz, CDCl_3_) δ 13.89 (s, 1H, OH), 8.39 (s, 1H, CH), 7.41 (s, 1H, ArH), 7.11 (s, 1H, ArH), 6.91 (d, *J* = 15.0 Hz, 5H, ArH), 6.86–6.82 (t, 4H, ArH), 6.73 (s, 1H, ArH), 5.07 (s, 1H, NH), 4.57 (s, 2H, CH_2_), 3.88 (d, *J* = 12.5 Hz, 2H, CH_2_), 3.79–3.69 (m, 34H, 8OCH_3_, 5CH_2_), 3.60–3.55 (d, J = 4 Hz, 2H), 2.45 (s, 2H, CH_2_), 1.82 (s, 2H, CH_2_), 1.59 (s, 4H, CH_2_), 1.49 (t, J = 6 Hz, 9H, CH_3_), 1.34 (d, *J* = 2.4 Hz, 9H, CH_3_), 1.02–0.98 (m, 3H, CH_3_), 0.86 (s, 2H, CH_2_), −0.07 (s, 2H, CH_2_), −1.29 (s, 4H, CH_2_), −2.32 (s, 2H, CH_2_); ^13^C NMR (Supplementary Figure S14) (101 MHz, CDCl_3_) δ 167.3, 165.5, 158.1, 150.8, 150.3, 150.2, 150.1, 150.0, 147.0, 140.1, 136.8, 129.5, 129.1, 128.3, 128.2, 128.0, 127.9, 127.2, 126.9, 125.6, 117.8, 114.9, 114.0, 113.6, 113.4, 112.7, 112.4, 68.0, 65.9, 60.3, 55.6, 55.4, 55.3, 55.1, 38.1, 35.1, 32.0, 31.5, 30.2, 29.4, 29.3, 28.8, 28.6, 28.3, 26.3, 23.6, 19.6, 14.1; HRMS (ESI) (Supplementary Figure S15) calcd. for C_72_H_94_N_2_O_12_([M + Na]^+^): 1201.6704, found: 1201.6683.

**6g**: yellow solid, yield 58%. m.p. 126–127°C; ^1^H NMR (Supplementary Figure S19) (400 MHz, CDCl_3_) δ 13.59 (s, 1H, OH), 8.30 (s, 1H, CH), 7.40 (d, *J* = 10.0 Hz, 2H, ArH), 6.98–6.77 (m, 10H, ArH), 6.71 (s, 1H, ArH), 5.06 (s, 1H, NH), 4.56 (s, 2H, CH_2_), 3.75 (m, 38H, 8OCH_3_, 7CH_2_), 2.45 (s, 2H, CH_2_), 1.87–1.79 (m, 2H, CH_2_), 1.72 (m, 2H, CH_2_), 1.57 (t, *J* = 7.4 Hz, 2H, CH_2_), 1.38 (m, 2H, CH_2_), 1.18 (m, 2H, CH_2_), 1.02 (t, *J* = 7.3 Hz, 3H, CH_3_), 0.77 (s, 2H, CH_2_), −0.08 (s, 2H, CH_2_), −1.36 (s, 4H, CH_2_), −2.35 (s, 2H, CH_2_); ^13^C NMR (Supplementary Figure S20) (101 MHz, CDCl_3_) δ 167.3, 163.4, 160.4, 150.8, 150.4, 150.3, 150.1, 130.0, 147.0, 134.9, 133.2, 129.4, 129.0, 128.3, 128.2, 128.1, 128.0, 127.8, 127.2, 126.8, 120.1, 119.1, 114.7, 113.9, 113.6, 113.3, 112.7, 112.4, 119.9, 67.8, 65.9, 59.7, 55.5, 55.4, 55.3, 55.1, 38.0, 32.1, 31.2, 30.8, 30.6, 30.2, 29.7, 29.3, 28.9, 28.7, 28.3, 28.1, 26.5, 23.6, 19.7, 14.1; HRMS (ESI) (Supplementary Figure S21) calcd. for C_66_H_81_BrN_2_O_12_([M + Na]^+^): 1195.4871, found: 1195.4853.

**6h**: yellow solid, yield 63%. m.p. 102–103°C; ^1^H NMR (Supplementary Figure S22) (400 MHz, CDCl_3_) δ 13.94 (s, 1H, OH), 8.39 (s, 1H, CH), 7.40 (d, *J* = 2.4 Hz, 1H, ArH), 7.11 (d, *J* = 2.4 Hz, 1H, ArH), 6.95–6.81 (m, 9H, ArH), 6.74 (d, *J* = 10.9 Hz, 1H, ArH), 5.12 (s, 1H, NH), 4.57 (s, 2H, CH_2_), 3.90–3.61 (m, 38H, 8OCH_3_, 7CH_2_), 2.51 (s, 2H, CH_2_), 1.83 (d, *J* = 14.9 Hz, 2H, CH_2_), 1.73 (t, *J* = 7.7 Hz, 2H, CH_2_), 1.61 (t, *J* = 7.6 Hz, 2H, CH_2_), 1.48 (s, 9H, CH_3_), 1.44–1.38 (t, J = 12 Hz, 2H, CH_2_), 1.32 (s, 9H, CH_3_), 1.18 (s, 2H, CH_2_), 1.02 (t, *J* = 7.4 Hz, 3H, CH_3_), 0.75 (s, 2H, CH_2_), −0.14 (s, 2H, CH_2_), −1.20–1.47 (t, *J* = 52 Hz, 4H, CH_2_), −2.36 (s, 2H, CH_2_); ^13^C NMR (Supplementary Figure S23) (101 MHz, CDCl_3_) δ 167.3, 165.7, 158.1, 150.9, 150.3, 150.3, 150.2, 150.0, 147.0, 140.1, 136.8, 129.3, 129.0, 128.4, 128.2, 128.1, 128.0, 127.9, 127.8, 127.2, 126.8, 125.6, 117.8, 114.7, 114.1, 114.0, 113.6, 113.3, 112.7, 112.7, 112.3, 67.8, 65.8, 59.6, 55.4, 55.4, 55.4, 55.3, 55.2, 55.1, 38.0, 35.1, 32.1, 31.5, 31.4, 30.8, 30.7, 30.1, 29.7, 29.4, 29.2, 28.9, 28.6, 28.5, 28.3, 28.2, 26.6, 23.6, 19.6, 14.1; HRMS (ESI) (Supplementary Figure S24) calcd. for C_74_H_98_N_2_O_12_([M + Na]^+^): 1229.7017, found: 1229.6999.

**7a**: white solid, yield 36%. m.p. 186–187°C; ^1^H NMR (Supplementary Figure S25) (400 MHz, CDCl_3_) δ 11.51 (s, 1H, NH), 7.89 (d, *J* = 6.6 Hz, 2H, ArH), 7.42 (t, *J* = 8 Hz, 3H, ArH), 6.94–6.82 (m, 9H, ArH), 6.72 (s, 1H, ArH), 5.71 (s, 1H, CH), 5.05 (s, 1H, NH), 4.57 (s, 2H, CH_2_), 3.89 (m, 2H, CH_2_), 3.80–3.71 (m, 34H, 8OCH_3_, 5CH_2_), 3.32 (m, 2H, CH_2_), 2.44 (s, 2H, CH_2_), 2.13 (s, 3H, CH_3_), 1.83 (m, 2H, CH_2_), 1.59–1.48 (m, 4H, CH_2_), 1.01 (t, *J* = 7.4 Hz, 3H, CH_3_), 0.87 (s, 2H, CH_2_), −0.03 (s, 2H, CH_2_), −1.37 (d, *J* = 60.8 Hz, 4H, CH_2_), −2.32 (s, 2H, CH_2_); ^13^C NMR (Supplementary Figure S26) (101 MHz, CDCl_3_) δ 187.6, 164.2, 150.9, 150.5, 150.4, 150.2, 150.0, 147.2, 140.4, 130.5, 129.7, 129.1, 128.4, 128.2, 128.1, 128.0, 127.9, 127.3, 126.8, 115.2, 114.3, 113.9, 113.7, 112.8, 112.5, 91.9, 68.3, 65.9, 55.8, 55.6, 55.4, 55.3, 55.2, 43.8, 38.0, 32.1, 31.1, 30.1, 29.3, 29.1, 28.9, 28.6, 28.3, 27.9, 19.7, 19.3, 14.1; MS (m/z): HRMS (ESI) (Supplementary Figure S27) calcd. for C_67_H_82_N_2_O_12_([M + Na]^+^): 1129.5765, found: 1129.5748.

**7b**: white solid, yield 36%. m.p. 212–213°C; ^1^H NMR (Supplementary Figure S28) (400 MHz, CDCl_3_) δ 11.53 (s, 1H, NH), 7.91–7.86 (t, *J* = 5.6 Hz, 2H, ArH), 7.42 (t, *J* = 6.4 Hz, 3H, ArH), 6.94–6.82 (m, 9H, ArH), 6.71 (s, 1H, ArH), 5.71 (s, 1H, CH), 5.04 (s, 1H, NH), 4.57 (s, 2H, CH_2_), 3.88 (t, *J* = 6.6 Hz, 2H, CH_2_), 3.80–3.71 (m, 34H, 8OCH_3_, 5CH_2_), 3.38 (m, 2H, CH_2_), 2.43 (s, 2H, CH_2_), 2.12 (s, 3H, CH_3_), 1.83 (s, 2H, CH_2_), 1.70 (t, *J* = 7.8 Hz, 2H, CH_2_), 1.62–1.57 (t, *J* = 7.2 Hz, 2H, CH_2_), 1.44 (t, *J* = 7.8 Hz, 2H, CH_2_), 1.20 (t, *J* = 7.7 Hz, 2H, CH_2_), 1.03 (t, *J* = 7.4 Hz, 3H, CH_3_), 0.81 (s, 2H, CH_2_), −0.05 (s, 2H, CH_2_), −1.39 (d, *J* = 47.7 Hz, 4H, CH_2_), −2.36 (s, 2H, CH_2_); ^13^C NMR (Supplementary Figure S29) (101 MHz, CDCl_3_) δ 187.7, 167.2, 164.6, 150.8, 150.4, 150.3, 150.2, 150.1, 150.0, 147.0, 140.4, 130.5, 129.4, 129.1, 128.6, 128.4, 128.2, 128.0, 127.9, 127.8, 127.1, 127.0, 126.8, 114.8, 114.0, 113.7, 113.3, 112.8, 112.4, 92.0, 77.3, 67.9, 55.5, 55.4, 55.3, 55.1, 43.4, 38.0, 32.1, 30.8, 30.7, 30.4, 30.2, 29.7, 29.3, 28.8, 28.7, 27.8, 19.7, 19.4, 14.1; MS (m/z): HRMS (ESI) (Supplementary Figure S30) calcd. for C_69_H_86_N_2_O_12_([M + Na]^+^): 1157.6078, found: 1157.6054.

**8a**: white solid, yield 54%. m.p. 87–88°C; ^1^H NMR (Supplementary Figure S31) (400 MHz, CDCl_3_) δ 10.86 (s, 1H, NH), 6.94–6.81 (m, 9H, ArH), 6.71 (s, 1H, ArH), 5.07 (s, 1H, NH), 5.00 (s, 1H, CH), 4.57 (s, 2H, CH_2_), 3.88 (t, *J* = 6.6 Hz, 2H, CH_2_), 3.81–3.67 (m, 34H, 8OCH_3_, 5CH_2_), 3.21 (m, 2H, CH_2_), 2.45 (s, 2H, CH_2_), 2.04 (s, 3H, CH_3_), 1.98 (s, 3H, CH_3_), 1.82 (m, 2H, CH_2_), 1.62–1.58 (t, *J* = 7.6 Hz, 2H, CH_2_), 1.42 (t, *J* = 7.8 Hz, 2H, CH_2_), 1.02 (t, *J* = 7.3 Hz, 3H, CH_3_), 0.79 (s, 2H, CH_2_), −0.08 (s, 2H, CH_2_), −1.32 (s, 4H, CH_2_), −2.33 (s, 2H, CH_2_); ^13^C NMR (Supplementary Figure S32) (101 MHz, CDCl_3_) δ 194.8, 167.2, 162.4, 150.9, 150.5, 150.4, 150.3, 150.2, 150.1, 150.0, 147.2, 129.6, 129.1, 128.4, 128.3, 128.2, 128.1, 128.0, 127.9, 127.3, 126.9, 115.7, 115.2, 114.3, 113.9, 113.7, 113.6, 112.8, 112.4, 95.1, 68.2, 55.7, 55.6, 55.5, 55.4, 55.3, 55.1, 43.6, 38.0, 32.05, 31.2, 29.3, 29.1, 28.8, 28.5, 28.3, 27.8, 19.7, 18.7, 14.1; MS (m/z): HRMS (ESI) (Supplementary Figure S33) calcd. for C_62_H_80_N_2_O_12_([M + Na]^+^): 1067.5609, found: 1067.5613.

**8b**: white solid, yield 39%. m.p. 86–87°C; ^1^H NMR (Supplementary Figure S34) (400 MHz, CDCl_3_) δ 10.92 (s, 1H, NH), 6.94–6.82 (m, 9H, ArH), 6.70 (s, 1H, ArH), 5.04 (s, 1H, NH), 5.00 (s, 1H, CH), 4.57 (s, 2H, CH_2_), 3.87 (t, *J* = 6.5 Hz, 2H, CH_2_), 3.80–3.68 (m, 34H, 8OCH_3_, 5CH_2_), 3.28 (m, 2H, CH_2_), 2.42 (s, 2H, CH_2_), 2.03 (s, 3H, CH_3_), 1.97 (s, 3H, CH_3_), 1.83 (s, 2H, CH_2_), 1.64 (d, *J* = 7.4 Hz, 2H, CH_2_), 1.58 (d, *J* = 7.4 Hz, 2H, CH_2_), 1.42–1.36 (m, 2H, CH_2_), 1.20–1.15 (t, *J* = 8 Hz, 2H, CH_2_), 1.03 (t, *J* = 7.4 Hz, 3H, CH_3_), 0.83–0.77 (d, *J* = 8.8 Hz, 2H,CH_2_), −0.06 (s, 2H, CH_2_), −1.40 (d, *J* = 53.4 Hz, 4H, CH_2_), −2.37 (s, 2H, CH_2_); ^13^C NMR (Supplementary Figure S35) (101 MHz, CDCl_3_) δ 194.9, 167.2, 162.9, 150.8, 150.4, 150.3, 150.2, 150.1, 150.0, 147.0, 129.4, 129.0, 128.4, 128.2, 128.2, 128.0, 127.9, 127.8, 127.1, 126.8, 114.8, 114.0, 113.7, 113.3, 112.7, 112.4, 95.2, 67.9, 65.9, 55.5, 55.4, 55.3, 55.1, 43.1, 38.0, 32.1, 30.8, 30.6, 30.5, 30.2, 29.7, 29.3, 28.8, 28.8, 28.7, 28.2, 27.8, 26.4, 19.7, 18.8, 14.1; MS (m/z): HRMS (ESI) (Supplementary Figure S36) calcd. for C_64_H_84_N_2_O_12_([M + Na]^+^): 1095.5922, found: 1095.5912.

**9a**: red solid, yield 56%. m.p. 100–102°C; ^1^H NMR (Supplementary Figure S37) (400 MHz, CDCl_3_) δ 8.45 (s, 1H, CH), 8.39 (m, 2H, ArH), 8.09–7.98 (m, 5H,ArH), 7.10 (d, *J* = 9.0 Hz, 1H, ArH), 6.94–6.89 (t, *J* = 14.4 Hz, 4H, ArH), 6.84 (d, *J* = 5.5 Hz, 4H, ArH), 6.74 (s, 1H, ArH), 5.15 (s, 1H, NH), 4.57 (s, 2H, CH_2_), 3.89 (s, 2H, CH_2_), 3.80–3.72 (m, 34H, 8OCH_3_, 5CH_2_), 3.55 (s, 2H, CH_2_), 2.53 (s, 2H,CH_2_), 1.82 (s, 2H, CH_2_), 1.61–1.57 (t, *J* = 7.6 Hz, 2H,CH_2_), 1.47 (s, 2H, CH_2_), 1.00 (t, *J* = 7.4 Hz, 3H, CH_3_), 0.76 (s, 2H, CH_2_), −0.11 (s, 2H, CH_2_), −1.18 (d, *J* = 62.5 Hz, 4H, CH_2_), −2.16 (s, 2H, CH_2_); ^13^C NMR (Supplementary Figure S38) (101 MHz, CDCl_3_) δ 196.3, 168.4, 167.4, 164.1, 156.0, 150.8, 150.5, 150.4, 150.3, 150.2, 150.0, 148.2, 147.2, 144.6, 130.6, 129.6, 129.1, 128.4, 128.3, 128.2, 128.1, 127.9, 127.4, 127.2, 127.0, 124.8, 124.8, 123.3, 123.0, 119.7, 119.0, 117.6, 115.1, 114.2, 114.0, 113.8, 113.6, 112.8, 112.4, 77.2, 68.2, 55.9, 55.8, 55.6, 55.5, 55.3, 55.1, 38.1, 32.1, 31.4, 30.2, 29.3, 28.9, 28.3, 27.8, 19.6, 14.1; MS (m/z): HRMS (ESI) (Supplementary Figure S39) calcd. for C_70_H_81_N_5_O_14_([M + Na]^+^): 1238.5678, found: 1238.5674.

**9b**: red solid, yield 89%. m.p. 100–101°C; ^1^H NMR (Supplementary Figure S40) (400 MHz, CDCl_3_) δ 8.46 (s, 1H, CH), 8.37 (d, *J* = 8.5 Hz, 2H, ArH), 8.06 (d, *J* = 9.4 Hz, 1H, ArH), 7.98 (d, *J* = 8.6 Hz, 3H, ArH), 7.09 (d, *J* = 9.0 Hz, 1H, ArH), 6.94–6.83 (m, 9H, ArH), 6.72 (s, 1H, ArH), 5.08 (s, 1H, NH), 4.57 (s, 2H, CH_2_), 3.88 (s, 2H, CH_2_), 3.75 (m, 34H, 8OCH3, 5CH_2_), 3.68 (s, 2H, CH_2_), 2.47 (s, 2H, CH_2_), 1.79 (d, *J* = 33.9 Hz, 4H, CH_2_), 1.60 (d, *J* = 7.5 Hz, 2H, CH_2_), 1.41 (s, 2H, CH_2_), 1.20 (s, 2H, CH_2_), 1.02 (t, *J* = 7.5 Hz, 3H, CH_3_), 0.80 (s, 2H, CH_2_), −0.06 (s, 2H, CH_2_), −1.31 (s, 4H, CH_2_), −2.31 (s, 2H, CH_2_); ^13^C NMR (Supplementary Figure S41) (101 MHz, CDCl_3_) δ 164.3, 156.0, 150.8, 150.4, 150.3, 150.2, 150.1, 150.0, 148.2, 147.0, 144.6, 129.6, 129.4, 129.0, 128.3, 128.2, 128.0, 127.9, 127.8, 127.2, 126.8, 124.7, 123.3, 123.0, 119.6, 117.6, 114.8, 114.0, 113.7, 113.3, 112.7, 112.4, 77.2, 67.9, 58.2, 55.5, 55.4, 55.3, 55.1, 38.0, 32.1, 31.0, 30.8, 30.5, 30.2, 29.7, 29.3, 28.8, 28.6, 28.3, 28.0, 23.6, 19.6, 14.1; MS (m/z): HRMS (ESI) (Supplementary Figure S42) calcd. for C_70_H_81_N_5_O_14_([M + Na]^+^): 1266.5991, found: 1266.5969.

### Synthesis of Monomers

Synthesis of compound **Monomer-1**: methyl 2-(4-butoxyphenoxy) acetate (1 g, 4.2 mmol) and 1,10-decanediamine (7.7 g, 44.8 mmol) were added to 20 ml of anhydrous ethanol solution and reacted at 75°C for 12 h. The organic solvent was removed by rotation under reduced pressure, and compound **Monomer-1** was obtained by column chromatography (volume ratio: dichloromethane: methanol = 10: 1). White solid, 60%; ^1^H NMR (Supplementary Figure S43) (400 MHz, CDCl_3_) δ 6.84 (s, 4H, ArH), 6.65 (s, 1H, NH_2_), 5.31 (s, 1H, NH), 4.43 (s, 2H, CH_2_), 3.91 (t, *J* = 6.5 Hz, 2H, CH_2_), 3.69 (m, 2H, CH_2_), 3.36–3.30 (m, 2H, CH_2_), 1.75–1.72 (m, 6H, CH_2_), 1.51–1.40 (m, 10H, CH_2_), 1.23 (t, *J* = 7.0 Hz, 4H, CH_2_), 0.97 (t, *J* = 7.4 Hz, 3H, CH_3_).

Synthesis of compound **Monomer-2**: **Monomer-1** (0.208 g, 0.55 mmol) and salicylaldehyde (0.06 g, 0.5 mmol) were added to 20 ml of anhydrous ethanol solution and reacted at 80°C for 4 h. The organic solvent was removed by rotating under reduced pressure, and the compound **Monomer-2** was obtained by column chromatography (volume ratio: ethyl acetate: petroleum ether = 1:5). Yellow solid, 50%; ^1^H NMR (Supplementary Figure S44) (400 MHz, CDCl_3_) δ 13.73 (s, 1H, OH), 8.33 (s, 1H, CH), 7.32–7.27 (m, 1H, ArH), 6.95 (d, *J* = 8.2 Hz, 1H, ArH), 6.84 (s, 4H, ArH), 6.60 (s, 1H, ArH), 4.43 (s, 2H, CH_2_), 3.91 (t, *J* = 6.5 Hz, 2H, CH_2_), 3.58 (t, *J* = 6.9 Hz, 2H, CH_2_), 3.33 (m, 2H, CH_2_), 1.79–1.64 (m, 4H, CH_2_), 1.49 (m, 6H, CH_2_), 1.28 (t, *J* = 4.9 Hz, 10H, CH_2_), 0.97 (t, *J* = 7.4 Hz, 3H, CH_3_).

### Materials and Methods

Stoppers **2a**, **2c**, **2d**, **3**, **4**, and **5** and reagents [1,10-decanediamine, methyl 2-(4-butoxyphenoxy)acetate, and so on] were commercially available (99%) and used as received. Further purification and drying of the solvents by standard methods were employed and distilled prior to use when necessary.

^1^H NMR and ^13^C NMR spectra were recorded on a Bruker AVIII-400 MHz spectrometer. 2D NMR spectra were recorded on a Bruker AV-600 MHz spectrometer. All NMR used tetramethylsilane (TMS) as the internal standard.

A Bruker Micro-TOF spectrometer was used to investigate the high-resolution mass (ESI) of the compounds.

A Bruker Smart APEX-2 CCD diffractometer was used to investigate the X-ray single-crystal structures.

## Results and Discussion

### ^1^H NMR Investigation

**FIGURE 1:**
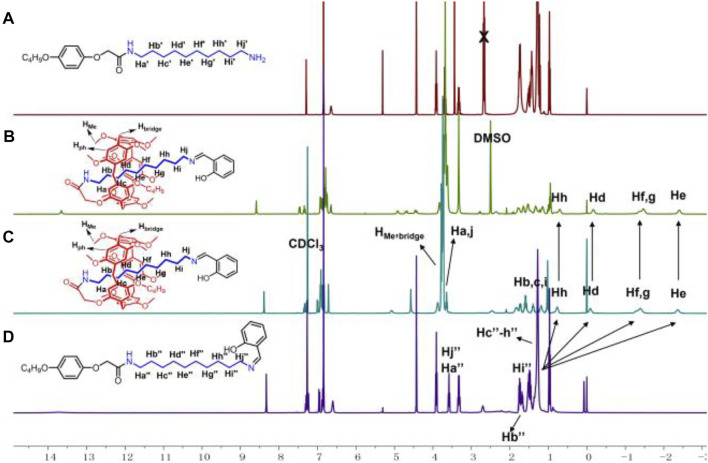
^1^H NMR spectra (400 MHz, 298K) of **(A) monomer-1** in CDCl_3_, **(B) 6e** in DMSO-d_6_, **(C) 6e** in CDCl_3_, and **(D) monomer-2** in CDCl_3_.

As shown in Scheme 2, **P5[1]Rs** were synthesized from pseudo[1]rotaxane **1a** or **1b** reacted with different stoppers in one step. We used ^1^H NMR spectra to characterize the obtained **P5[1]Rs** firstly. It can be clearly found that there were several groups of protons in the high magnetic field (δ < 0 ppm) of the ^1^H NMR spectra of **6a**, **6c**, **6d**, **6e**, **6g**, **6h**, **7a**, **7b**, **8a**, **8b**, **9a**, and **9b**, indicating that the alkyl chain was penetrated into the cavity of pillar[5]arene (Zhang et al., 2020; Ding et al., 2021). [1]Rotaxane **6e** is taken as an example; **monomer-2** and **6e** in CDCl_3_ at 293 K are shown in Figures 1D,C. Compared with **monomer-2**, we found that the signals of protons Hd, He, Hf, Hg, and Hh on the alkyl chain attached onto the pillar[5]arene platform shifted upfield from 1.277 to 0.756, −0.094, −1.292, −1.382, and −2.401 ppm due to the shielding effect, indicating the formation of a mechanically interlocked structure. Then, we used DMSO-*d*_6_ as the solvent for ^1^H NMR investigation to confirm the formation of [1]rotaxane. As is known that DMSO is a de-complexometric solvent, in DMSO-*d*_6_, the signals of protons on the alkyl chains upfield obviously below 0 ppm due to the shielding effect (Figure 1B), confirming that the stopper units are large enough for blocking the cavity of pillar[5]arene. Further ^13^C NMR and HR-MS studies also confirmed that these **P5[1]Rs** were prepared successfully.

### 2D NOESY Investigation

**FIGURE 2:**
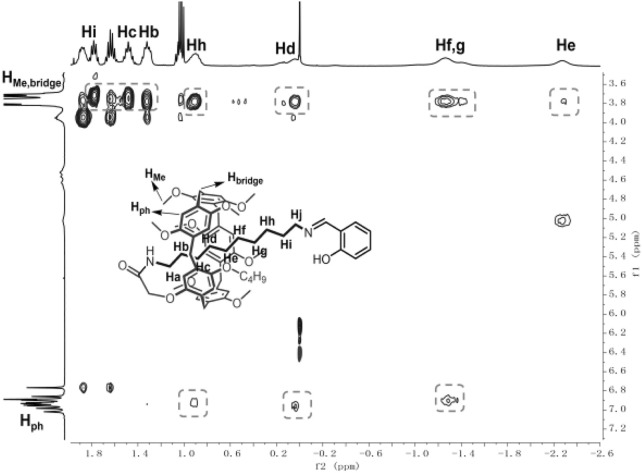
Partial 2D NOESY spectrum of a CDCl_3_ solution of **6e** at 298K.

2D nuclear overhauser effect spectroscopy (NOESY) was then used to characterize the stereochemical structure of the obtained [1]rotaxanes. We also take **6e** as an example. As shown in Figure 2, the hydrogens (Hb, Hc, Hd, He, Hf, Hg, Hh, and Hi) of the alkyl chain on **6e** showed strong correlation with the bridged -CH_2_- (H_bridge_) and the hydrogen–OCH_3_ and -OCH_2_- (H_Me_) on the 1,4-dimethoxybenzene, indicating that the alkyl chain was passed through the cavity and consisted of the above ^1^H NMR results. The NOESY spectrum of 6e in DMSO-*d*_6_ also confirmed the interlocked structure (Supplementary Figure S45).

### Single-Crystal Structures

**FIGURE 3:**
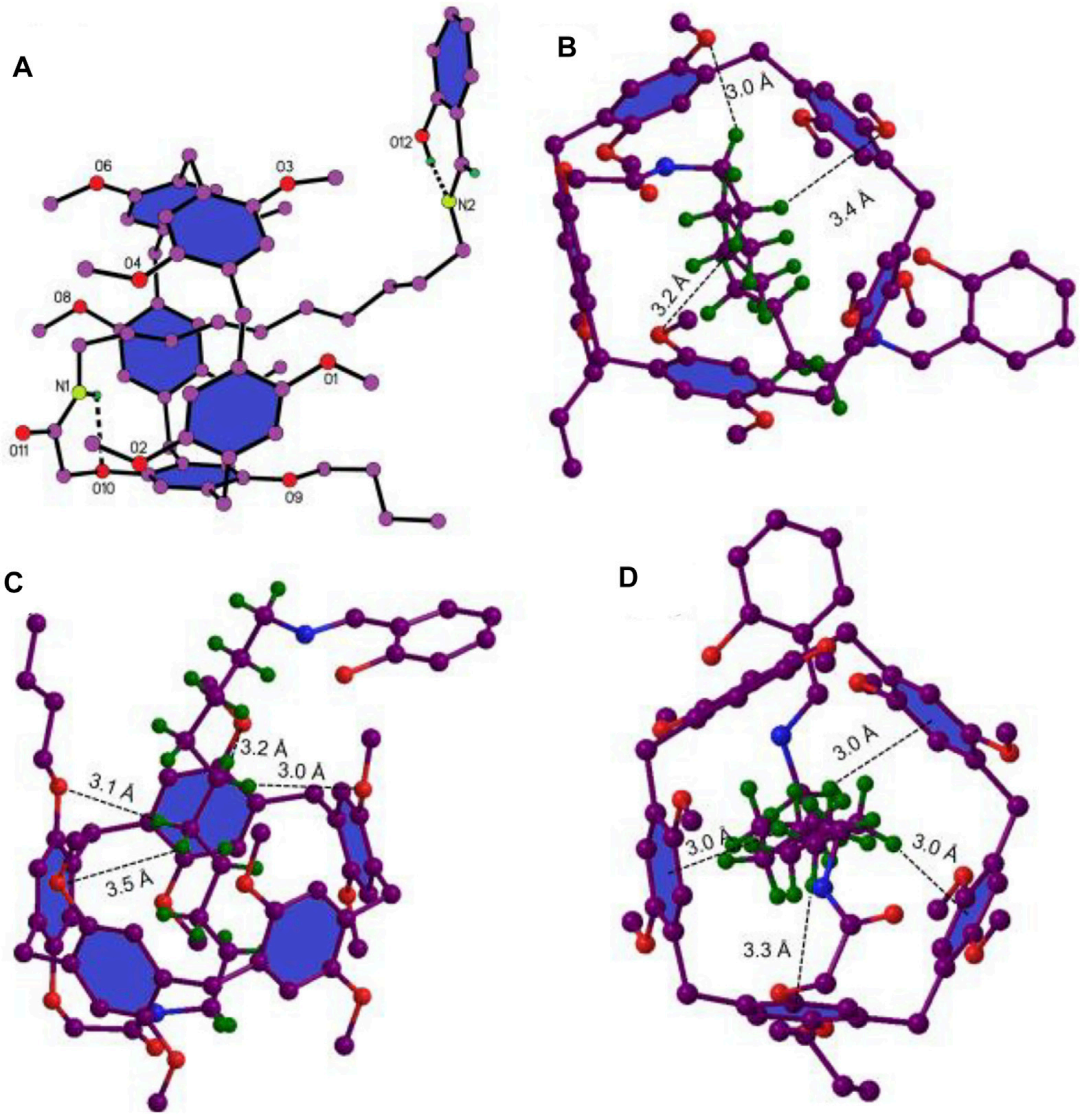
The single-crystal structure of **6e**, where only the hydrogens in question are given for clarity. **(A)** N–H···O and O–H···N hydrogen bonding. **(B)** and **(C)** C–H···O interaction, H···O distances, 3.0 Å, 3.2 Å, 3.4 Å, 3.0 Å, 3.2 Å, 3.1 Å, and 3.5 Å. **(D)** C–H···π interactions, H···ring center distances, 3.0 Å, 3.3 Å 3.0 Å, and 3.0 Å.

With the aim to further study the mechanically interlocked structure of **6e**, a colorless crystal was grown by a vapor diffusion method. As shown in Figure 3A, the alkyl chain penetrated into the cavity of pillar[5]arene and the salicylaldiminato unit acted as a stopper to form [1]rotaxane. One amide N–H···O hydrogen bonding and an O–H···N hydrogen bonding were observed in Figure 3A. Besides the weak C–H···O interactions between the CH_2_ of the axle and the O atoms of the host (Figures 3B,C), multiple C–H···π interactions between CH_2_ of the axle and benzene units of the host (Figure 3D) were also observed. So, the driving forces for the formation of [1]rotaxane was the synergic C–H···π, C–H···O interactions and N–H···O hydrogen bonding.

## Conclusion

In this article, we have successfully designed and synthesized three series of pillar [5]arene-based [1]rotaxanes (**P5[1]Rs**) with pentanedione derivatives, azobenzene derivatives, and salicylaldehyde derivatives as the stoppers, respectively. ^1^H NMR, ^13^C NMR, 2D-NOESY, MS, and single-crystal X-ray analysis were used to characterize the obtained **P5[1]Rs**. We found that the driving forces for the formation of [1]rotaxane were the synergic C–H···π, C–H···O interactions and N–H···O, O–H···N hydrogen bonding. This work not only enriched the diversity of pillar[n]arene family but also gave a big boost to the pillar[n]arene-based MIMs.

## Data Availability

The data presented in the study are deposited in the Cambridge Crystallographic Data Centre repository, accession number 2120389.
