# Supplementary material for: Construction and Property Investigation of Serial Pillar[5]arene-Based [1]Rotaxanes
Source: Front Chem. 2022 Jun 7;10:908773. doi: 10.3389/fchem.2022.908773 (PMC9210957; doi:10.3389/fchem.2022.908773)
Supplement: Supplementary file 1 [file DataSheet3.zip › ╘¡╩╝╩2╛▌╒√└φ/2D NOESY-6e/1/pdata/1/email_20211118FC0016MLT-1_1_1.pdf]

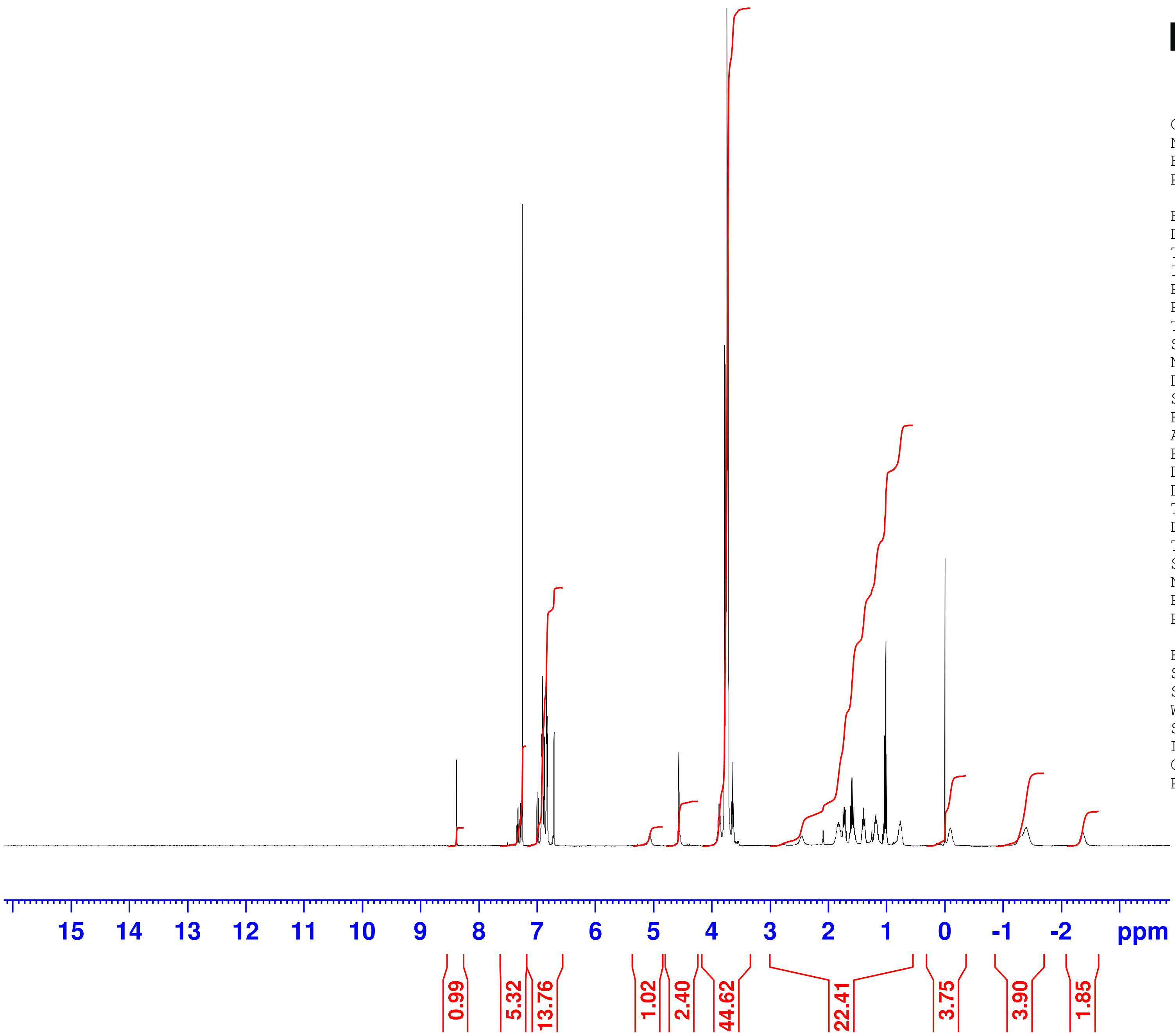

Current Data Parameters
NAME 20211118FC0016MLT-1
EXPNO 1
PROCNO 1

F2 - Acquisition Parameters

Date_	20211118
Time	17.32 h
INSTRUM	spect
PROBHD	Z116098_0503 (
PULPROG	zg30
TD	65536
SOLVENT	CDCl3
NS	16
DS	2
SWH	8012.820 Hz
FIDRES	0.122266 Hz
AQ	4.0894465 sec
RG	86.5
DW	62.400 usec
DE	6.50 usec
TE	296.8 K
D1	1.00000000 sec
TD0	1
SFO1	400.1324708 MHz
NUC1	1H
P1	9.70 usec
PLW1	15.02099991 W

F2 - Processing parameters

SI	65536
SF	400.1300104 MHz
WDW	EM
SSB	0
LB	0.30 Hz
GB	0
PC	1.00
