# Supplementary material for: Construction and Property Investigation of Serial Pillar[5]arene-Based [1]Rotaxanes
Source: Front Chem. 2022 Jun 7;10:908773. doi: 10.3389/fchem.2022.908773 (PMC9210957; doi:10.3389/fchem.2022.908773)
Supplement: Supplementary file 1 [file DataSheet3.zip › ╘¡╩╝╩2╛▌╒√└φ/2D NOESY-6e/2/pdata/1/email_20211118FC0016MLT-1_2_1.pdf]

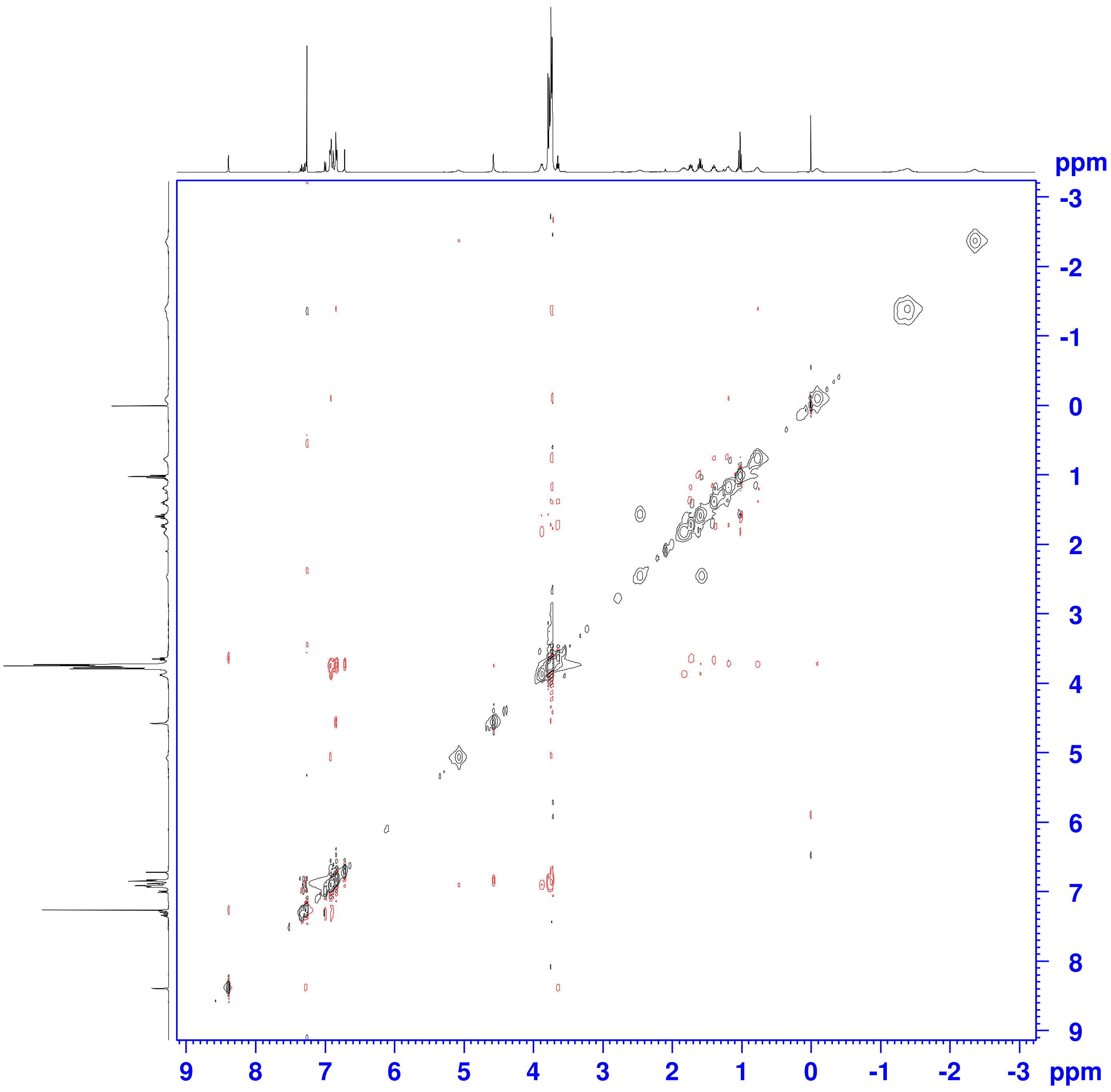


```
Current Data Parameters
NAME       20211118FC0016MLT-1
EXPNO                        2
PROCNO                       1

F2 - Acquisition Parameters
Date_               20211118
Time                   17.34 h
INSTRUM                spect
PROBHD     Z116098_0503 (
PULPROG         noesygpphpp
TD                      2048
SOLVENT                CDCl3
NS                         4
DS                        32
SWH                 4950.495 Hz
FIDRES              2.417234 Hz
AQ                 0.2068480 sec
RG                     50.36
DW                   101.000 usec
DE                      6.50 usec
TE                     296.8 K
D0                0.00008865 sec
D1                2.04915190 sec
D8                0.30000001 sec
D11               0.03000000 sec
D12               0.00002000 sec
D16               0.00020000 sec
IN0               0.00020200 sec
TDav                       1
SFO1             400.1311910 MHz
NUC1                      1H
P1                      9.70 usec
P2                     19.40 usec
P17                  2500.00 usec
PLW1             15.02099991 W
PLW10             1.57040000 W
GPNAM[1]          SMSQ10.100
GPZ1                   40.00 %
P16                  1000.00 usec

F1 - Acquisition parameters
TD                       256
SFO1                400.1312 MHz
FIDRES             19.337872 Hz
SW                    12.372 ppm
FnMODE            States-TPPI

F2 - Processing parameters
SI                      1024
SF               400.1300104 MHz
WDW                    QSINE
SSB                        2
LB              0 Hz
GB              0
PC                      1.00

F1 - Processing parameters
SI                      1024
MC2               States-TPPI
SF               400.1300104 MHz
WDW                    QSINE
SSB                        2
LB              0 Hz
GB              0
```
