# Supplementary material for: Construction and Property Investigation of Serial Pillar[5]arene-Based [1]Rotaxanes
Source: Front Chem. 2022 Jun 7;10:908773. doi: 10.3389/fchem.2022.908773 (PMC9210957; doi:10.3389/fchem.2022.908773)
Supplement: Supplementary file 1 [file DataSheet3.zip › ╘¡╩╝╩2╛▌╒√└φ/H--6e--δ«┤·DMSO/pdata/1/email_20210917FC0006MLT-1_1_1.pdf]

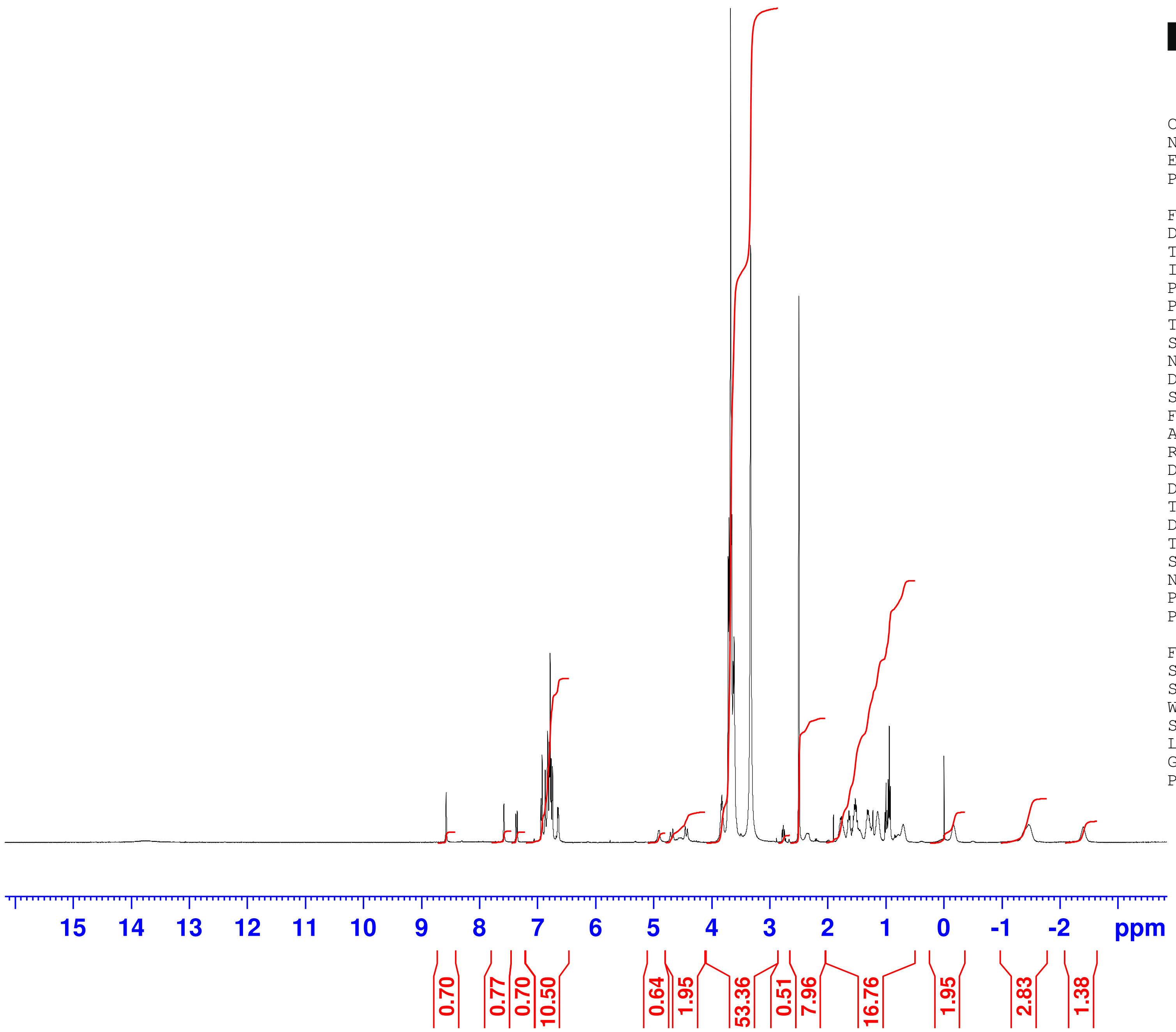

Current Data Parameters
NAME 20210917FC0006MLT-1
EXPNO 1
PROCNO 1

F2 - Acquisition Parameters
Date_ 20210918
Time 7.36 h
INSTRUM spect
PROBHD Z116098_0503 (
PULPROG zg30
TD 65536
SOLVENT DMSO
NS 16
DS 2
SWH 8012.820 Hz
FIDRES 0.122266 Hz
AQ 4.0894465 sec
RG 77.33
DW 62.400 usec
DE 6.50 usec
TE 296.6 K
D1 1.00000000 sec
TD0 1
SFO1 400.1324708 MHz
NUC1 1H
P1 9.70 usec
PLW1 15.02099991 W

F2 - Processing parameters
SI 65536
SF 400.1300022 MHz
WDW EM
SSB 0
LB 0.30 Hz
GB 0
PC 1.00
