# Supplementary material for: Construction and Property Investigation of Serial Pillar[5]arene-Based [1]Rotaxanes
Source: Front Chem. 2022 Jun 7;10:908773. doi: 10.3389/fchem.2022.908773 (PMC9210957; doi:10.3389/fchem.2022.908773)

checkCIF/PLATON report

You have not supplied any structure factors. As a result the full set of tests cannot be run.

THIS REPORT IS FOR GUIDANCE ONLY. IF USED AS PART OF A REVIEW PROCEDURE FOR PUBLICATION, IT SHOULD NOT REPLACE THE EXPERTISE OF AN EXPERIENCED CRYSTALLOGRAPHIC REFEREE.

No syntax errors found. CIF dictionary Interpreting this report

Datablock: LD20201222_a

Bond precision: C-C = 0.0040 A Wavelength=0.71073

Cell:	a=12.4222(9)	b=34.267(3)	c=14.3191(11)
	alpha=90	beta=101.218(2)	gamma=90
Temperature:	296 K		

	Calculated	Reported
Volume	5978.8(8)	5978.8(8)
Space group	P 21/c	P 21/c
Hall group	-P 2ybc	-P 2ybc
Moiety formula	C66 H82 N2 O12	C66 H82 N2 O12
Sum formula	C66 H82 N2 O12	C66 H82 N2 O12
Mr	1095.34	1095.33
Dx,g cm-3	1.217	1.217
Z	4	4
Mu (mm-1)	0.083	0.083
F000	2352.0	2352.0
F000'	2353.13	
h,k,lmax	14,40,17	14,40,17
Nref	10518	10494
Tmin,Tmax	0.980,0.985	0.691,0.746
Tmin'	0.980	

Correction method= # Reported T Limits: Tmin=0.691 Tmax=0.746
AbsCorr = MULTI-SCAN

Data completeness= 0.998 Theta(max)= 24.999

R(reflections)= 0.0882(4560) wR2(reflections)= 0.2808(10494)

S = 0.995 Npar= 731

The following ALERTS were generated. Each ALERT has the format
test-name_ALERT_alert-type_alert-level.
Click on the hyperlinks for more details of the test.

Alert level B

PLAT097_ALERT_2_B Large Reported Max. (Positive) Residual Density 0.81 eA-3
PLAT220_ALERT_2_B NonSolvent Resd 1 C Ueq(max)/Ueq(min) Range 7.6 Ratio
PLAT230_ALERT_2_B Hirshfeld Test Diff for O3 --C17 . 10.0 s.u.
PLAT230_ALERT_2_B Hirshfeld Test Diff for O5 --C22 . 9.0 s.u.
PLAT230_ALERT_2_B Hirshfeld Test Diff for O8 --C34 . 7.5 s.u.
PLAT230_ALERT_2_B Hirshfeld Test Diff for O8 --C36 . 7.3 s.u.
PLAT230_ALERT_2_B Hirshfeld Test Diff for O9 --C44 . 8.0 s.u.
PLAT230_ALERT_2_B Hirshfeld Test Diff for O12 --C66 . 10.0 s.u.
PLAT230_ALERT_2_B Hirshfeld Test Diff for C31 --C32 . 8.5 s.u.
PLAT230_ALERT_2_B Hirshfeld Test Diff for C38 --C39 . 7.5 s.u.
PLAT230_ALERT_2_B Hirshfeld Test Diff for C44 --C45 . 9.7 s.u.
PLAT230_ALERT_2_B Hirshfeld Test Diff for C56 --C57 . 8.3 s.u.
PLAT230_ALERT_2_B Hirshfeld Test Diff for C57 --C58 . 9.3 s.u.
PLAT230_ALERT_2_B Hirshfeld Test Diff for C61 --C62 . 10.3 s.u.
PLAT241_ALERT_2_B High 'MainMol' Ueq as Compared to Neighbors of C65 Check
PLAT936_ALERT_2_B The Embedded .res File Includes a DAMP Command . 1000.0 Report

Alert level C

DIFMN02_ALERT_2_C The minimum difference density is < -0.1*ZMAX*0.75
_refine_diff_density_min given = -0.615
Test value = -0.600
DIFMN03_ALERT_1_C The minimum difference density is < -0.1*ZMAX*0.75
The relevant atom site should be identified.
DIFMX02_ALERT_1_C The maximum difference density is > 0.1*ZMAX*0.75
The relevant atom site should be identified.
PLAT026_ALERT_3_C Ratio Observed / Unique Reflections (too) Low .. 43% Check
PLAT084_ALERT_3_C High wR2 Value (i.e. > 0.25) .... 0.28 Report
PLAT098_ALERT_2_C Large Reported Min. (Negative) Residual Density -0.62 eA-3
PLAT220_ALERT_2_C NonSolvent Resd 1 O Ueq(max)/Ueq(min) Range 5.9 Ratio
PLAT222_ALERT_3_C NonSolvent Resd 1 H Uiso(max)/Uiso(min) Range 9.1 Ratio
PLAT230_ALERT_2_C Hirshfeld Test Diff for O3 --C13 . 6.0 s.u.
PLAT230_ALERT_2_C Hirshfeld Test Diff for O9 --C40 . 6.2 s.u.
PLAT230_ALERT_2_C Hirshfeld Test Diff for N2 --C60 . 5.2 s.u.
PLAT230_ALERT_2_C Hirshfeld Test Diff for C14 --C15 . 7.0 s.u.
PLAT230_ALERT_2_C Hirshfeld Test Diff for C15 --C16 . 6.5 s.u.
PLAT230_ALERT_2_C Hirshfeld Test Diff for C20 --C21 . 7.0 s.u.
PLAT230_ALERT_2_C Hirshfeld Test Diff for C20 --C25 . 7.0 s.u.
PLAT230_ALERT_2_C Hirshfeld Test Diff for C33 --C34 . 6.0 s.u.
PLAT230_ALERT_2_C Hirshfeld Test Diff for C38 --C43 . 6.5 s.u.
PLAT230_ALERT_2_C Hirshfeld Test Diff for C60 --C61 . 6.5 s.u.
PLAT230_ALERT_2_C Hirshfeld Test Diff for C64 --C65 . 5.1 s.u.
PLAT234_ALERT_4_C Large Hirshfeld Difference C61 --C66 . 0.19 Ang.
PLAT234_ALERT_4_C Large Hirshfeld Difference C62 --C63 . 0.18 Ang.
PLAT234_ALERT_4_C Large Hirshfeld Difference C65 --C66 . 0.18 Ang.
PLAT241_ALERT_2_C High 'MainMol' Ueq as Compared to Neighbors of C59 Check
PLAT241_ALERT_2_C High 'MainMol' Ueq as Compared to Neighbors of C64 Check
PLAT242_ALERT_2_C Low 'MainMol' Ueq as Compared to Neighbors of C56 Check
PLAT242_ALERT_2_C Low 'MainMol' Ueq as Compared to Neighbors of C61 Check
PLAT242_ALERT_2_C Low 'MainMol' Ueq as Compared to Neighbors of C66 Check

Alert level G

```
PLAT002_ALERT_2_G Number of Distance or Angle Restraints on AtSite         22 Note
PLAT003_ALERT_2_G Number of Uiso or Uij Restrained non-H Atoms ...         10 Report
PLAT007_ALERT_5_G Number of Unrefined Donor-H Atoms .............           2 Report
PLAT172_ALERT_4_G The CIF-Embedded .res File Contains DFIX Records         14 Report
PLAT174_ALERT_4_G The CIF-Embedded .res File Contains FLAT Records          1 Report
PLAT177_ALERT_4_G The CIF-Embedded .res File Contains DELU Records          1 Report
PLAT178_ALERT_4_G The CIF-Embedded .res File Contains SIMU Records          1 Report
PLAT860_ALERT_3_G Number of Least-Squares Restraints ............         113 Note
PLAT883_ALERT_1_G No Info/Value for _atom_sites_solution_primary .     Please Do !
PLAT933_ALERT_2_G Number of OMIT Records in Embedded .res File ...          9 Note
```

```
   0 ALERT level A = Most likely a serious problem - resolve or explain
  16 ALERT level B = A potentially serious problem, consider carefully
  27 ALERT level C = Check. Ensure it is not caused by an omission or oversight
  10 ALERT level G = General information/check it is not something unexpected

   3 ALERT type 1 CIF construction/syntax error, inconsistent or missing data
  38 ALERT type 2 Indicator that the structure model may be wrong or deficient
   4 ALERT type 3 Indicator that the structure quality may be low
   7 ALERT type 4 Improvement, methodology, query or suggestion
   1 ALERT type 5 Informative message, check
```

It is advisable to attempt to resolve as many as possible of the alerts in all categories. Often the minor alerts point to easily fixed oversights, errors and omissions in your CIF or refinement strategy, so attention to these fine details can be worthwhile. In order to resolve some of the more serious problems it may be necessary to carry out additional measurements or structure refinements. However, the purpose of your study may justify the reported deviations and the more serious of these should normally be commented upon in the discussion or experimental section of a paper or in the "special_details" fields of the CIF. checkCIF was carefully designed to identify outliers and unusual parameters, but every test has its limitations and alerts that are not important in a particular case may appear. Conversely, the absence of alerts does not guarantee there are no aspects of the results needing attention. It is up to the individual to critically assess their own results and, if necessary, seek expert advice.

Publication of your CIF in IUCr journals

A basic structural check has been run on your CIF. These basic checks will be run on all CIFs submitted for publication in IUCr journals (*Acta Crystallographica*, *Journal of Applied Crystallography*, *Journal of Synchrotron Radiation*); however, if you intend to submit to *Acta Crystallographica Section C* or *E* or *IUCrData*, you should make sure that full publication checks are run on the final version of your CIF prior to submission.

Publication of your CIF in other journals

Please refer to the *Notes for Authors* of the relevant journal for any special instructions relating to CIF submission.

PLATON version of 13/07/2021; check.def file version of 13/07/2021

Datablock LD20201222_a - ellipsoid plot

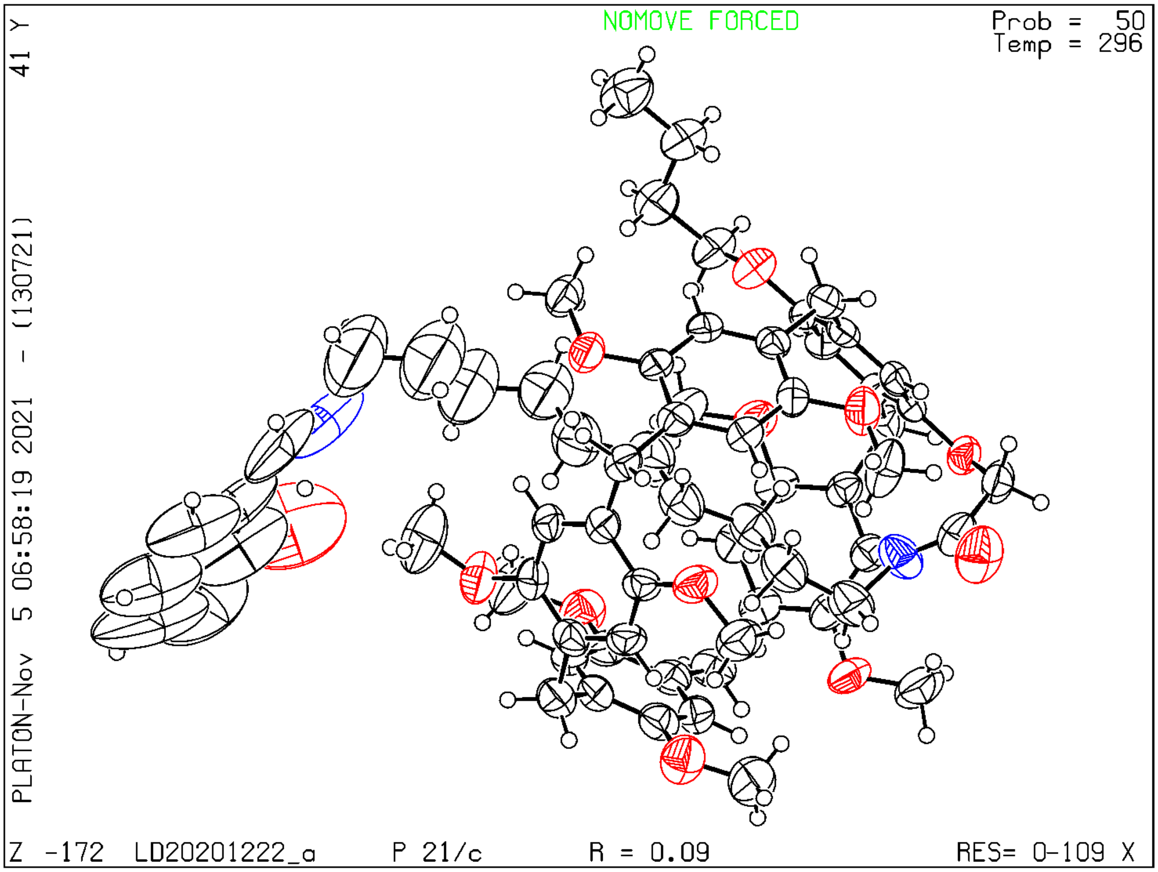

Supplement: Supplementary file 2 [file DataSheet4.zip › ╛o╠σ ╓∙[5]+10C╢■░╖+╦«╤ε╚⌐/checkcif.pdf]
