# Supplementary material for: Construction and Property Investigation of Serial Pillar[5]arene-Based [1]Rotaxanes
Source: Front Chem. 2022 Jun 7;10:908773. doi: 10.3389/fchem.2022.908773 (PMC9210957; doi:10.3389/fchem.2022.908773)
Supplement: Supplementary file 4 [file DataSheet1.docx]

**Construction and property investigation of a serials pillar[5]arene-based [1]rotaxanes**

Longtao Ma^1,2^, Ying Han^1^*, Chaoguo Yan^1^, Tingting Chen^2^, Yang Wang^2^, and Yong Yao^2^*

^1^School of Chemistry and Chemical Engineering, Yangzhou University, Yangzhou, Jiangsu, 225001, P.R. China

^2^School of Chemistry and Chemical Engineering, Nantong University, Nantong, Jiangsu, 226019, P.R. China

Hanying@yzu.edu.cn; yaoyong1986@ntu.edu.cn

**Supporting Information (26 pages)**

| 1. | Materials and methods | S2 |
| --- | --- | --- |
| 2. | NMR spectra of **1a** and **1b** | S3 |
| 3. | Characterizations of [1]rotaxenes | S6 |
| 4. | Characterizations of Monomers | S25 |

1. **Materials and methods**

All reagents and solvents were commercially available in analytical grade and used as received. Further purification and drying by standard methods were employed and distilled prior to use when necessary. All evaporations of organic solvents were carried out with a rotary evaporator in conjunction with a water aspirator.

NMR spectra were collected on either a Bruker AVIII-400 MHz spectrometer or a Bruker AV-600 MHz spectrometer with internal standard tetramethylsilane (TMS) and signals as internal references, and the chemical shifts (δ) were expressed in ppm.

High-resolution Mass (ESI) spectra were obtained with a Bruker Micro-TOF spectrometer.

X-ray data were collected on a Bruker Smart APEX-2 CCD diffractometer.

1. **NMR spectra of 1a and 1b**^S1^

**1a**: White solid, 50%, m.p.155-157℃; ^1^H NMR (400 MHz, CDCl_3_) δ 6.93 – 6.82 (m, 9H, ArH), 6.74 (d, *J* = 7.7 Hz, 1H, ArH), 5.19 (s, 1H, NH), 4.57 (s, 2H, CH_2_), 3.89 (m, 3H, CH_2_), 3.80 – 3.71 (m, 34H, 8OCH_3_, 5CH_2_), 3.63 (s, 1H, CH_2_), 2.66 (t, *J* = 7.2 Hz, 2H, CH_2_), 1.88 – 1.81 (m, 2H, CH_2_), 1.61 (m, 2H, CH_2_), 1.23 (m, 2H, CH_2_), 1.04 (t, *J* = 7.4 Hz, 3H, CH_3_), 0.69 (m, 2H, CH_2_), -0.12 (s, 2H, CH_2_), -1.15 (s, 2H, CH_2_), -1.39 (s, 2H, CH_2_), -2.22 (s, 2H, CH_2_); ^13^C NMR (101 MHz, CDCl_3_) δ 167.3, 150.8, 150.4, 150.4, 150.3, 150.3, 150.2, 150.0, 147.0, 129.4, 129.1, 128.4, 128.3, 128.2, 128.1, 128.0, 127.8, 127.3, 126.9, 114.9, 114.1, 113.9, 113.5, 112.8, 112.7, 112.5, 112.4, 68.0, 66.0, 55.7, 55.6, 55.5, 55.4, 55.3, 55.1, 42.8, 38.0, 34.8, 32.1, 30.1, 29.3, 29.2, 28.9, 28.6, 28.5, 28.4, 27.7, 26.7, 23.7, 19.6, 14.1; IR(KBr) υ: 3404.05, 2932.50, 2855.13, 2482.04, 2145.84, 2039.04, 1678.90, 1610.10, 1500.35, 1464.81, 1399.98, 1306.58, 1213.24, 1100.97, 1047.92, 928.69, 878.14, 854.71, 774.01, 704.73, 647.36, 604.78, 541.10, 454.15 cm^-1^; MS (m/z): HRMS (ESI) Calcd. for C_57_H_74_N_2_O_11_([M + H]^+^): 963.5371, found: 963.5392.

**Figure S1**. ^1^H NMR spectra (400 MHz, 298K, CDCl_3_) of **1a**.

**Figure S2**. ^13^C NMR spectra (101 MHz, 298K, CDCl_3_) of **1a**.

**Figure S3**. HR-MS spectra of **1a**. Calcd. for C_57_H_74_N_2_O_11_([M + H]^+^): 963.5371, found: 963.5392.

**1b**: White solid, 40%, m.p.130-131℃; ^1^H NMR (400 MHz, CDCl_3_) δ 6.94 – 6.82 (m, 9H, ArH), 6.72 (s, 1H, ArH), 5.10 (s, 1H, NH), 4.57 (s, 2H, CH_2_), 3.88 (m, 3H, CH_2_), 3.82 – 3.70 (m, 34H, 8OCH_3_, 5CH_2_), 3.64 (s, 1H, CH_2_), 2.73 (t, *J* = 7.1 Hz, 2H, CH_2_), 1.84 (t, *J* = 7.5 Hz, 2H, CH_2_), 1.62 – 1.57 (t, J = 7.6 Hz, 2H, CH_2_), 1.49 – 1.45 (t, J = 7.6 Hz, 2H, CH_2_), 1.32 (d, *J* = 7.2 Hz, 2H, CH_2_), 1.16 (d, *J* = 8.0 Hz, 2H, CH_2_), 1.04 (t, *J* = 7.4 Hz, 3H, CH_3_), 0.77 (s, 2H, CH_2_), -0.06 (s, 2H, CH_2_), -1.31 (s, 4H, CH_2_), -2.29 (s, 2H, CH_2_); ^13^C NMR (101 MHz, CDCl_3_) δ 167.2, 150.8, 150.7, 150.4, 150.3, 150.1, 150.1, 150.0, 147.0, 129.4, 129.0, 128.3, 128.2, 128.1, 128.0, 127.8, 127.1, 126.8, 114.7, 114.0, 113.9, 113.6, 113.2, 112.8, 112.7, 112.5, 112.4, 67.8, 65.9, 55.4, 55.3, 55.2, 55.1, 42.4, 38.0, 34.2, 32.1, 30.9, 30.8, 29.7, 29.2, 28.9, 28.7, 28.3, 27.8, 26.5, 23.6, 19.6, 14.1; IR(KBr) υ: 3407.95, 2930.79, 2853.95, 2146.30, 2039.48, 1680.45, 1499.90, 1464.94, 1399.77, 1304.96, 1213.17, 1101.25, 1048.14, 929.12, 878.58, 855.36, 774.36, 704.94, 647.41, 606.87, 541.77, 451.37 cm^-1^; MS (m/z): HRMS (ESI) Calcd. for C_59_H_78_N_2_O_11_([M + H]^+^): 991.5684, found: 991.5709.

**Figure S4**. ^1^H NMR spectra (400 MHz, 298K, CDCl_3_) of **1b**.

**Figure S5**. ^13^C NMR spectra (101 MHz, 298K, CDCl_3_) of **1b**.

**Figure S6**. HR-MS spectra of **1b**. Calcd. for C_59_H_78_N_2_O_11_([M + H]^+^): 991.5684, found: 991.5709.

1. **Characterizations of [1]rotaxenes**

**Figure S7**. ^1^H NMR spectra (400 MHz, 298K, CDCl_3_) of [1]rotaxene **6a**.

**Figure S8**. ^13^C NMR spectra (101 MHz, 298K, CDCl_3_) of [1]rotaxene **6a**.

**Figure S9**. HR-MS spectra of [1]rotaxene **6a**. Calcd. for C_64_H_78_N_2_O_12_([M + Na]^+^): 1089.5452, found: 1089.5437.

**Figure S10**. ^1^H NMR spectra (400 MHz, 298K, CDCl_3_) of [1]rotaxene **6c**.

**Figure S11**. ^13^C NMR spectra (101 MHz, 298K, CDCl_3_) of [1]rotaxene **6c**.

**Figure S12**. HR-MS spectra of [1]rotaxene **6c**. Calcd. for C_64_H_77_BrN_2_O_12_([M + Na]^+^): 1167.4558, found: 1167.4537.

**Figure S13**. ^1^H NMR spectra (400 MHz, 298K, CDCl_3_) of [1]rotaxene **6d**.

**Figure S14**. ^13^C NMR spectra (101 MHz, 298K, CDCl_3_) of [1]rotaxene **6d**.

**Figure S15**. HR-MS spectra of [1]rotaxene **6d**. Calcd. for C_72_H_94_N_2_O_12_([M + Na]^+^): 1201.6704, found: 1201.6683.

**Figure S16**. ^1^H NMR spectra (400 MHz, 298K, CDCl_3_) of [1]rotaxene **6e**.

**Figure S17**. ^13^C NMR spectra (101 MHz, 298K, CDCl_3_) of [1]rotaxene **6e**.

**Figure S18**. HR-MS spectra of [1]rotaxene **6e**. Calcd. for C_66_H_82_N_2_O_12_([M + Na]^+^): 1117.5765, found: 1117.5770.

**Figure S19**. ^1^H NMR spectra (400 MHz, 298K, CDCl_3_) of [1]rotaxene **6g**.

**Figure S20**. ^13^C NMR spectra (101 MHz, 298K, CDCl_3_) of [1]rotaxene **6g**.

**Figure S21**. HR-MS spectra of [1]rotaxene **6g**. Calcd. for C_66_H_81_BrN_2_O_12_([M+Na]^+^): 1195.4871, found: 1195.4853.

**Figure S22**. ^1^H NMR spectra (400 MHz, 298K, CDCl_3_) of [1]rotaxene **6h**.

**Figure S23**. ^13^C NMR spectra (101 MHz, 298K, CDCl_3_) of [1]rotaxene **6h**.

**Figure S24**. HR-MS spectra of [1]rotaxene **6h**. Calcd. for C_74_H_98_N_2_O_12_([M + Na]^+^): 1229.7017, found: 1229.6999.

**Figure S25**. ^1^H NMR spectra (400 MHz, 298K, CDCl_3_) of [1]rotaxene **7a**.

**Figure S26**. ^13^C NMR spectra (100 MHz, 298K, CDCl_3_) of [1]rotaxene **7a**.

**Figure S27**. HR-MS spectra of [1]rotaxene **7a**. Calcd. for C_67_H_82_N_2_O_12_([M + Na]^+^): 1129.5765, found: 1129.5748.

**Figure S28**. ^1^H NMR spectra (400 MHz, 298K, CDCl_3_) of [1]rotaxene **7b**.

**Figure S29**. ^13^C NMR spectra (100 MHz, 298K, CDCl_3_) of [1]rotaxene **7b**.

**Figure S30**. HR-MS spectra of [1]rotaxene **7b**. Calcd. for C_69_H_86_N_2_O_12_([M+Na]^+^): 1157.6078, found: 1157.6054.

**Figure S31**. ^1^H NMR spectra (400 MHz, 298K, CDCl_3_) of [1]rotaxene **8a**.

**Figure S32**. ^13^C NMR spectra (100 MHz, 298K, CDCl_3_) of [1]rotaxene **8a**.

**Figure S33**. HR-MS spectra of [1]rotaxene **8a**. C_62_H_80_N_2_O_12_([M + Na]^+^): 1067.5609, found: 1067.5613.

**Figure S34**. ^1^H NMR spectra (400 MHz, 298K, CDCl_3_) of [1]rotaxene **8b**.

**Figure S35**. ^13^C NMR spectra (100 MHz, 298K, CDCl_3_) of [1]rotaxene **8b**.

**Figure S36**. HR-MS spectra of [1]rotaxene **8b**. Calcd. for C_64_H_84_N_2_O_12_([M+Na]^+^): 1095.5922, found: 1095.5912.

**Figure S37**. ^1^H NMR spectra (400 MHz, 298K, CDCl_3_) of [1]rotaxene **9a**.

**Figure S38**. ^13^C NMR spectra (100 MHz, 298K, CDCl_3_) of [1]rotaxene **9a**.

**Figure S39**. HR-MS spectra of [1]rotaxene **9a**. Calcd. for C_70_H_81_N_5_O_14_([M + Na]^+^): 1238.5678, found: 1238.5674.

**Figure S40**. ^1^H NMR spectra (400 MHz, 298K, CDCl_3_) of [1]rotaxene **9b**.

**Figure S41**. ^13^C NMR spectra (100 MHz, 298K, CDCl_3_) of [1]rotaxene **9b**.

**Figure S42**. HR-MS spectra of [1]rotaxene **9b**. Calcd. for C_70_H_81_N_5_O_14_([M + Na]^+^): 1266.5991, found: 1266.5969.

Table S1. Single crystal data of **6e**

| Phase |  |
| --- | --- |
| Empirical formula | C_66_H_82_N_2_O_12_ |
| Formula weight | 1095.33 |
| Temperature(K) | 296(2) |
| Wavelength(Å) | 0.71073 |
| Crystal system,  space group | Monoclinic  P2(1)/c |
| a(Å) | 12.4222(9) |
| b(Å) | 34.267(3) |
| c(Å) | 14.3191(11) |
| α(°) | 90 |
| β(°) | 101.218(2) |
| γ(°) | 90 |
| Volume(Å^3^) | 5978.8(8) |
| Z | 4 |
| Calculated density(Mg·m^-3^) | 1.217 |
| Absorption coefficient(mm^-1^) | 0.083 |
| F(000) | 2352 |
| Crystal size(mm) | 0.240 x 0.220 x 0.180 |
| Theta range for data collection(°) | 1.774 to 24.999 |
| *hkl* ranges | -14 to 14, -40 to 40, -15 to 17 |
| Reflections collected  unique | 60520 / 10494  R(int)=0.1123 |
| Completeness to theta | 99.8% |
| Absorption correction | Semi-empirical from equivalents |
| Refinement method | Full-matrix least-squares on F^2^ |
| Data/restraints/parameters | 10494/113/731 |
| Goodness-of-fit on F^2^ | 0.995 |
| Final R indices [I>2σ(I)] | R_1_=0.0882,  wR_2_=0.2384 |
| R indices (all data) | R_1_=0.2107,  wR_2_=0.2808 |
| Largest diff. peak and Hole(e·Å^3^) | 0.811 and -0.615 |

1. **Characterization of the monomers**

**Monomer-1**: White solid, 60%; ^1^H NMR (400 MHz, CDCl_3_) δ 6.84 (s, 4H, ArH), 6.65 (s, 1H, NH_2_), 5.31 (s, 1H, NH), 4.43 (s, 2H, CH_2_), 3.91 (t, *J* = 6.5 Hz, 2H, CH_2_), 3.69 (m, 2H, CH_2_), 3.36 – 3.30 (m, 2H, CH_2_), 1.75 – 1.72 (m, 6H, CH_2_), 1.51 – 1.40 (m, 10H, CH_2_), 1.23 (t, *J* = 7.0 Hz, 4H, CH_2_), 0.97 (t, *J* = 7.4 Hz, 3H, CH_3_).

**Figure S43**. ^1^H NMR spectra (400 MHz, 298K, CDCl_3_) of **Monomer-1**.

**Monomer-2**: Yellow solid, 50%;^1^H NMR (400 MHz, CDCl_3_) δ 13.73 (s, 1H, OH), 8.33 (s, 1H, CH), 7.32 – 7.27 (m, 1H, ArH), 6.95 (d, *J* = 8.2 Hz, 1H, ArH), 6.84 (s, 4H, ArH), 6.60 (s, 1H, ArH), 4.43 (s, 2H, CH_2_), 3.91 (t, *J* = 6.5 Hz, 2H, CH_2_), 3.58 (t, *J* = 6.9 Hz, 2H, CH_2_), 3.33 (m, 2H, CH_2_), 1.79 – 1.64 (m, 4H, CH_2_), 1.49 (m, 6H, CH_2_), 1.28 (t, *J* = 4.9 Hz, 10H, CH_2_), 0.97 (t, *J* = 7.4 Hz, 3H, CH_3_).

**Figure S44**. ^1^H NMR spectra (400 MHz, 298K, CDCl_3_) of **Monomer-2**.


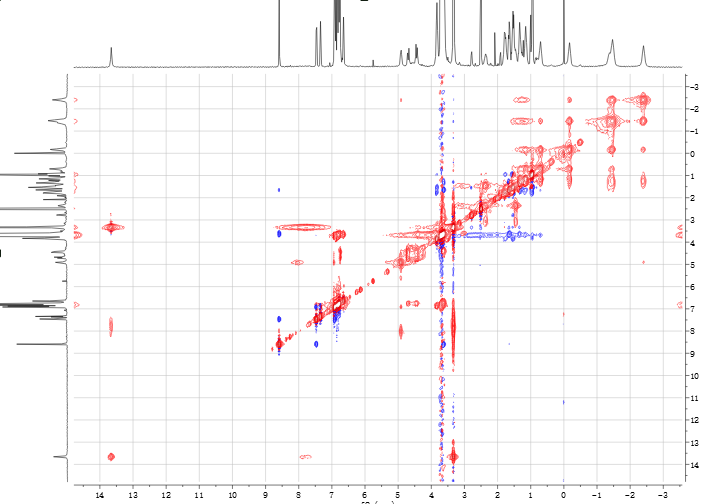


**Figure S45**. 2D NOESY spectrum of a DMSO-*d*_6_ solution of **6e** at 298K.

**References**

S1. Zhao, R., Wang, C., Long, R., Chen, T., Yan, C., and Yao, Y. (2019). Pillar[5]arene Based [1]rotaxane Systems With Redox-Responsive Host-Guest Property: Design, Synthesis and the Key Role of Chain Length. *Front. Chem.* 7, 508. doi: 10.3389/fchem.2019.00508.
