# Supplementary material for: Construction and Property Investigation of Serial Pillar[5]arene-Based [1]Rotaxanes
Source: Front Chem. 2022 Jun 7;10:908773. doi: 10.3389/fchem.2022.908773 (PMC9210957; doi:10.3389/fchem.2022.908773)
Supplement: Supplementary file 1 [file DataSheet3.zip › ╘¡╩╝╩2╛▌╒√└φ/H--Monomer-2/pdata/1/email_20220323001YY-MLT-1_1_1.pdf]

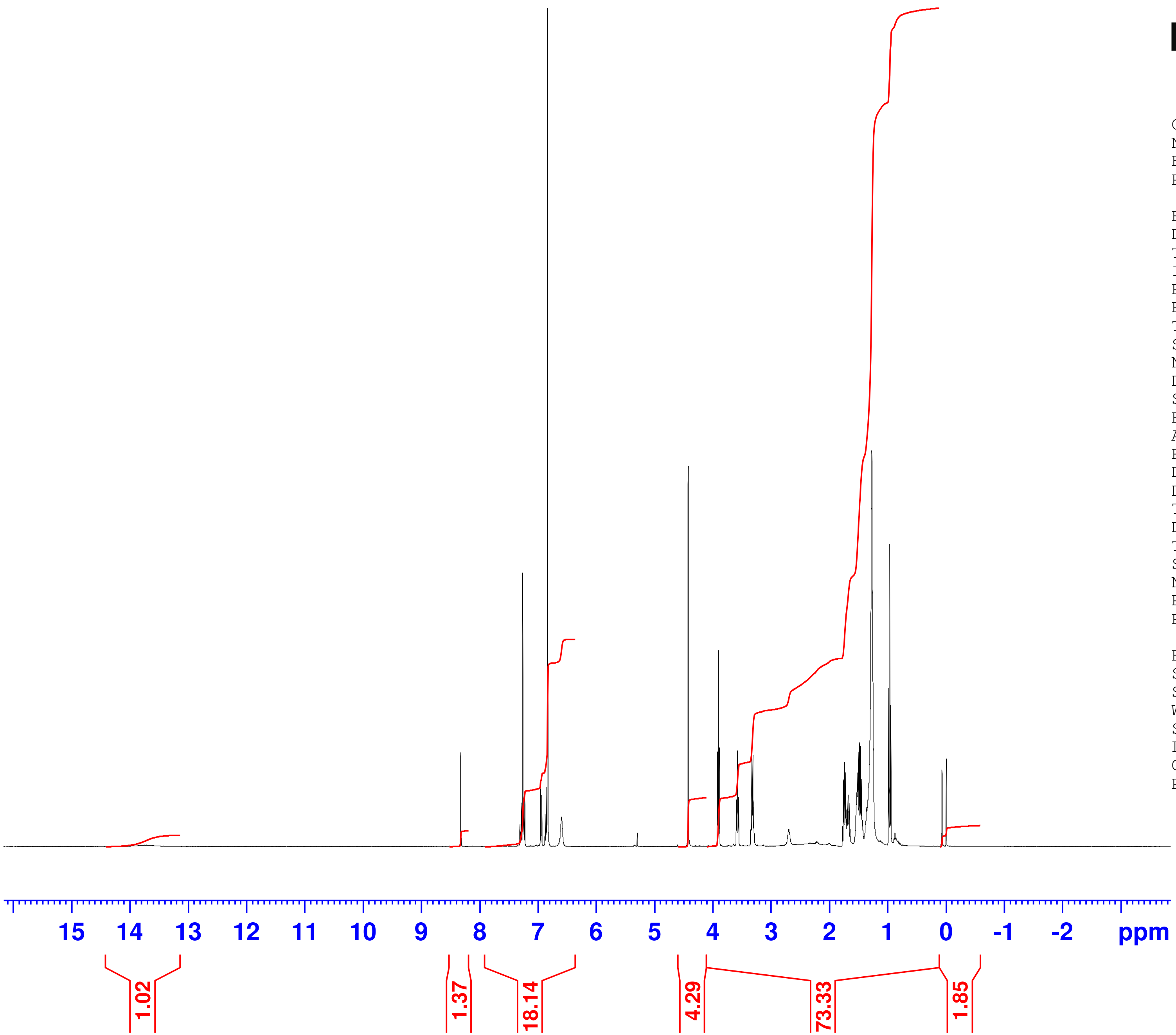

BRUKER

Current Data Parameters

NAME	20220323001YY-MLT-1
EXPNO	1
PROCNO	1

F2 - Acquisition Parameters

Date_	20220323
Time	9.43 h
INSTRUM	spect
PROBHD	Z116098_0503 (
PULPROG	zg30
TD	65536
SOLVENT	CDCl3
NS	16
DS	2
SWH	8012.820 Hz
FIDRES	0.122266 Hz
AQ	4.0894465 sec
RG	56.51
DW	62.400 usec
DE	6.50 usec
TE	291.1 K
D1	1.00000000 sec
TD0	1
SFO1	400.1324708 MHz
NUC1	1H
P1	9.70 usec
PLW1	15.02099991 W

F2 - Processing parameters

SI	65536
SF	400.1300074 MHz
WDW	EM
SSB	0
LB	0.30 Hz
GB	0
PC	1.00
