# Supplementary material for: Construction and Property Investigation of Serial Pillar[5]arene-Based [1]Rotaxanes
Source: Front Chem. 2022 Jun 7;10:908773. doi: 10.3389/fchem.2022.908773 (PMC9210957; doi:10.3389/fchem.2022.908773)
Supplement: Supplementary file 2 [file DataSheet4.zip › ╛o╠σ ╓∙[5]+10C╢■░╖+╦«╤ε╚⌐/═╝400ú¿ldú¿0201222_a.doc]

图400
